# One‐Step Biocatalytic Synthesis of Sustainable Surfactants by Selective Amide Bond Formation**

**DOI:** 10.1002/anie.202205054

**Published:** 2022-06-08

**Authors:** Max Lubberink, William Finnigan, Christian Schnepel, Christopher R. Baldwin, Nicholas J. Turner, Sabine L. Flitsch

**Affiliations:** ^1^ Department of Chemistry The University of Manchester Manchester Institute of Biotechnology 131 Princess Street Manchester M1 7DN UK

**Keywords:** Amides, Amino Alcohols, Biocatalysis, Surfactants, Sustainable Chemistry

## Abstract

*N*‐alkanoyl‐*N*‐methylglucamides (MEGAs) are non‐toxic surfactants widely used as commercial ingredients, but more sustainable syntheses towards these compounds are highly desirable. Here, we present a biocatalytic route towards MEGAs and analogues using a truncated carboxylic acid reductase construct tailored for amide bond formation (CAR*mm*‐A). CAR*mm*‐A is capable of selective amide bond formation without the competing esterification reaction observed in lipase catalysed reactions. A kinase was implemented to regenerate ATP from polyphosphate and by thorough reaction optimisation using design of experiments, the amine concentration needed for amidation was significantly reduced. The wide substrate scope of CAR*mm*‐A was exemplified by the synthesis of 24 commercially relevant amides, including selected examples on a preparative scale. This work establishes acyl‐phosphate mediated chemistry as a highly selective strategy for biocatalytic amide bond formation in the presence of multiple competing alcohol functionalities.

## Graphical abstract

An enzymatic route to commercially important surfactants is presented. A truncated construct of carboxylic acid reductase (CAR*mm*‐A) catalyzes amide bond formation between fatty acids and amino alcohols with no esterification observed. The wide substrate scope of the enzyme, co‐factor recycling, reaction engineering and up‐scaling show the feasibility of this method for synthesis.

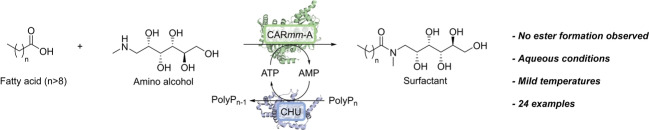

Surfactants are a ubiquitous class of compounds offering a wide range of applications in pharmaceuticals, cosmetics, detergents, emulsifiers, and foaming agents.[^1^, ^2^] As the global surfactant production is around 16.5 megatons per year, there is significant interest in developing surfactants from renewable resources and the use of more sustainable synthesis methods.[^3^, ^4^]

Non‐ionic surfactants like *N*‐alkanoyl‐*N*‐methylglucamides (MEGAs), are non‐toxic, stable, and biodegradable and are therefore widely utilized in pharmaceutical and biochemical applications.[^4^, ^5^] As current industrial syntheses towards MEGAs use harsh chemical methods, there has been a significant interest for alternative, more sustainable processes towards these targets, as exemplified by the recent “Sugar Surfactant Open Innovation Challenge” announced by the Swiss multinational company Clariant.^[6]^

To address this issue, enzymatic methods have gained some interest over the years. Lipase catalyzed synthesis of glucamide surfactants has been reported in either organic solvents or solvent‐free systems using high reaction temperatures.[^4^, ^7^] These lipase‐catalyzed reactions using *N*‐methyl‐d‐glucamine (**1**) and either a fatty acid or a fatty acid methyl ester yield a mixture of amide (target product) and ester by‐products, which can react with the acyl donor a second time to form an amide‐ester, which is reported to be the major by‐product.^[5]^

As amide bonds are prevalent motifs in many natural products and pharmaceuticals, there has been growing interest in the development of aqueous amidation methods under benign conditions.^[8]^ Biocatalysts, in particular ATP‐dependent enzymes, have gained increasing interest over recent years.[^9^, ^10^, ^11^, ^12^, ^13^, ^14^, ^15^, ^16^]

We have previously reported on using carboxylic acid reductase (CAR) for amide bond formation by intercepting the adenylate intermediate by substituting the NADPH cofactor for an amine nucleophile.^[17]^ Others have shown that CARs are also capable of lactam formation.^[18]^ Subsequently, it was found that using a truncated construct of CAR, consisting of a stand‐alone adenylation domain (CAR*mm*‐A), was more effective in amide bond formation, and optimization of this process allowed for the selective mono‐acylation of symmetrical diamines.^[19]^ A recent study showed that CARs are also capable of ester formation. However, significant amounts of imidazole catalyst and alcohol nucleophile concentrations of over 1000‐fold excess were required to observe ester formation.^[20]^

Encouraged by these results, we were interested in the question of whether CAR*mm*‐A could fill the need for the selective biocatalytic formation of *N*‐alkanoyl amide based surfactants as outlined in Figure 1. CARs are particularly attractive for this approach because of their wide substrate scope, which includes long‐chain fatty acids,^[21]^ whereas acyltransferases are limited to short‐chain acyl donors[^22^, ^23^] or are very selective towards one amino sugar.^[24]^

**Figure 1:**
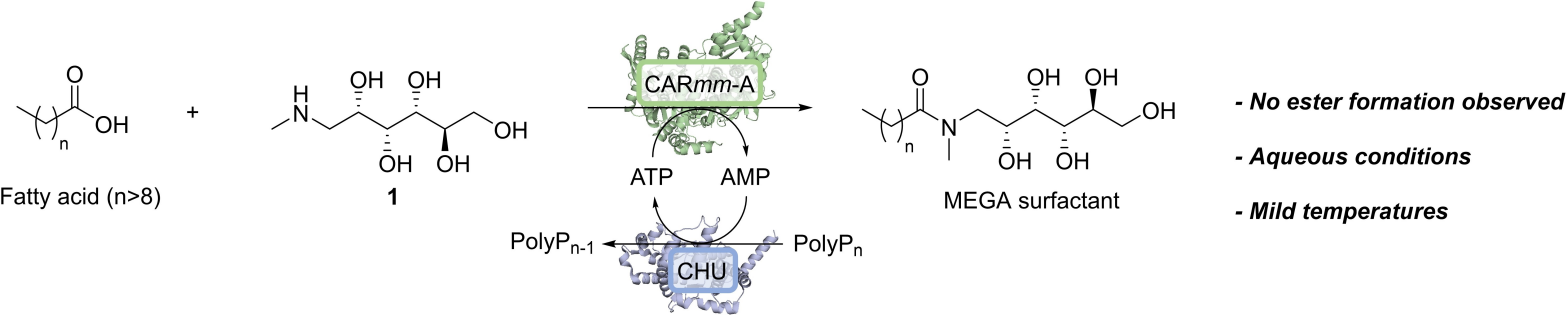
Selective CAR*mm*‐A catalysed synthesis of MEGA surfactants. The cofactor can be recycled in situ using the kinase CHU.

In initial efforts to test our hypothesis, *N*‐methylglucamine **1** was reacted with octanoic acid (**7**) using our previously described CAR*mm*‐A construct consisting of the adenylation domain of CAR*mm* from *Mycobacterium marinum*.^[19]^ Under previously reported conditions using an excess of amine and ATP, we obtained the target surfactant MEGA‐8 (**11**) in over 99 % conversion, as rigorously determined by RP‐HPLC at a wavelength of 210 nm, using a commercial standard as a reference (Figure S7, S8). The near quantitative conversion to product **11** suggested that amide bond formation was indeed highly selective over formation of any side‐products.

To provide additional experimental evidence for the observed selectivity of amide bond formation over ester formation, the reaction was further explored using isotopic labelling. The use of ^13^C‐labelled decanoic acid and **1** allowed us to monitor the reaction in situ by ^13^C NMR (Figure S1, S2). Importantly, only the desired amide ^13^C‐**13** was observed using this method, with no other carbonyl‐containing (ester) side product detected by NMR. Additionally, amidation between 3‐fluoro cinnamic acid and **1** allowed detection of substrates and reaction products by UV‐HPLC and ^19^F NMR. No side products were observed during this experiment (Figure S3–S5). Furthermore, using an excess of sorbitol as a nucleophile under these conditions did not lead to any ester products, further confirming the absence of ester formation by CAR*mm*‐A under these reaction conditions (Figure S6).

After these initial results, we first sought to address the stoichiometric use of ATP by implementing a polyphosphate kinase from *Cytophaga hutchinsonii* (CHU) to regenerate ATP from AMP. The use of CHU allows AMP to be utilized as a substrate rather than the more expensive ATP, with sodium polyphosphate as a phosphate source.[^19^, ^25^]

Secondly, the excess of amine over carboxylic acid should ideally be reduced, having previously required amine excess of up to 100‐fold.^[17]^ Pleasingly, reduction of amine (**1**) to 50 mM over 5 mM **9** still resulted in a good yield (78 %) of **13**, (Figure S10) suggesting that amino‐polyols are better substrates than previously described amines.^[17]^ Starting from these promising reaction conditions, design of experiments was used to construct an empirical model for the effect of polyphosphate, AMP and Mg^2+^ concentrations on conversion (Figure 2) with the view of further optimization.

**Figure 2:**
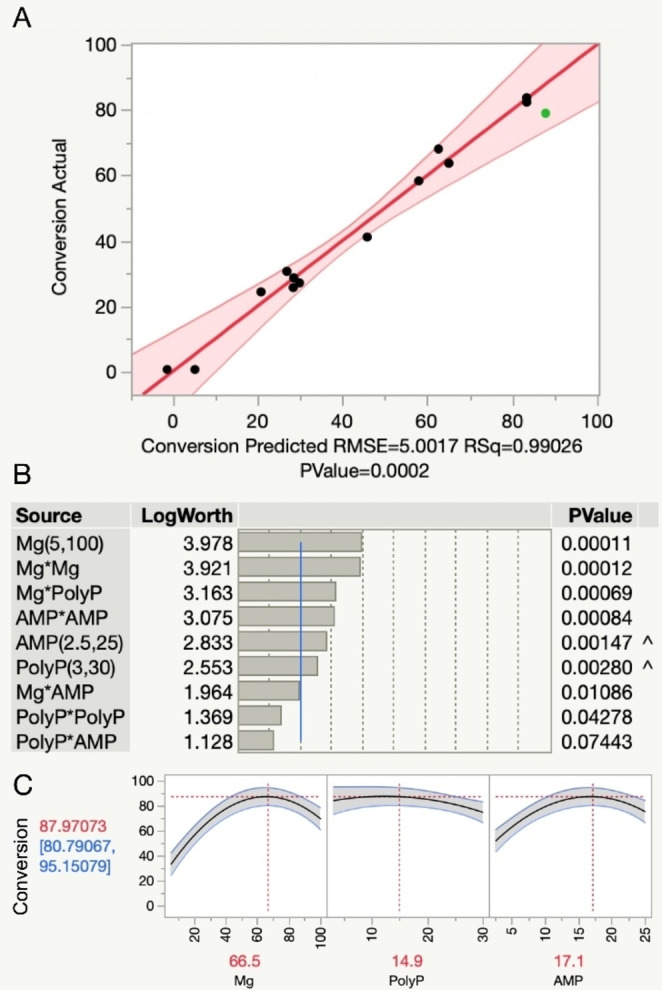
Empirical model of the reaction. A) The actual conversion plotted against the model prediction. The green data point represents a confirmation experiment using the optimized conditions. B) Pareto plots of model factor significance. C) Snapshot of the prediction profiler set to maximum desirability, showing the optimum concentrations of Mg^2+^, PolyP and AMP as predicted by the model.

Interestingly, magnesium concentration was the most important factor in the model, showing significant curvature and a strong interaction with polyphosphate concentration (Figure 2B). The model predicted that the optimum magnesium concentration went up as polyphosphate concentrations were increased. The shift in optimum magnesium concentration is likely due to chelation of magnesium ions by polyphosphate, sequestering them from ATP or AMP and therefore limiting conversion where this effect is too strong. This effect has been observed by others working on similar ATP regeneration systems.[^25^, ^26^, ^27^] Using the model predictions, optimal conditions of 17.1 mM AMP, 66.5 mM MgCl_2_, and 14.9 mg mL^−1^ polyphosphate were selected for future experiments (Figure 2C).

In further efforts to improve the system we observed that further reduction of amine concentration substantially lowered conversion (Figure S12). A second round of design of experiments was conducted to construct an empirical model for the interdependencies of acid, amine, and CAR*mm*‐A concentrations on conversion and calculated analytical yield (Figure S13). The main effects for acid, amine and CAR*mm*‐A concentrations were all significant, in addition to the quadratic effect for acid concentration and a small interaction between acid and amine concentrations. Counter‐intuitively, the model predicted yield to peak at 6 mM acid, decreasing with higher substrate loadings. We investigated whether increasing co‐solvent concentrations might be inhibiting the reaction but found DMSO concentrations up to 10 % to have only a minor effect on conversion and chose a DMSO concentration of 1 % in our reactions (Figure S14). We also ruled out any pH‐based effects at higher substrate loadings, suggesting some form of substrate inhibition could be causing the drop in yield at higher substrate loadings. The trade‐offs between yield, conversion, and the use of resources apparent in the model allow for exploration for what an optimized reaction might look like, depending on the desired process. We aimed to maximize yield while keeping amine concentration low, suggesting a 5 mM acid and 50 mM amine concentration using 2 mg mL^−1^ CAR*mm*‐A (0.55 mol %) to be optimal.

With these optimized conditions, we subsequently examined the scope of other amino alcohols for selective CAR*mm*‐A catalyzed amide formation using octanoic (**7**), nonanoic (**8**), decanoic (**9**), and dodecanoic acid (**10**) as acyl donors. It was found that like the secondary amine of **1** leading to amides **11**–**14** (MEGA‐8, MEGA‐9, MEGA‐10 and MEGA‐12 respectively), the primary amine of d‐glucamine (**2**) was also well accepted as amine donor forming amides **15**–**18** (Table 1). Further exploiting the *N*‐ selectivity of this system, we investigated the use of amino alcohols **3** and **4** as amine nucleophiles forming amides **19**–**26**. These ceramide analogues are widely used in the pharmaceutical and cosmetics industry but previously reported lipase‐catalyzed syntheses encounter ester side‐product formation as outlined before.^[28]^ We observed conversion for both amino alcohols, but the primary amine substrate **3** (conversions up to 83 %) performing significantly better than the secondary amine substrate **4** (conversions up to 39 %). Finally, we decided to look at ethanolamine (**5**) and diethanolamine (**6**) as amine donors, as their amide products would yield commercially available anti‐foaming agents such as lauramide MEA and DEA (**30** and **34** respectively). We found **5** to be a good amine donor in this system (conversions up to 77 %), but secondary amine **6** showed very poor conversions (up to 11 %) with dodecanoic acid product **34** not being observed at all. In general, fatty acid carbon chain lengths of up to 10 carbons were well accepted in this system, but a drop‐off in conversion was observed when dodecanoic acid was used as acyl donor, which is likely caused due to the limited solubility of dodecanoic acid in water.

**Table 1:** Synthesis of MEGA surfactants and surfactant‐like molecules using CAR*mm*‐A.

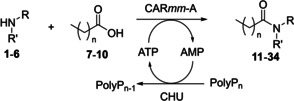					
		Conversion^[a]^			
Amine	Product	**7** *n*=6	**8** *n*=7	**9** *n*=8	**10** *n*=10
**1**	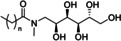	**11** 94 %	**12** 83 %	**13** 79 %	**14** 30 %
**2**	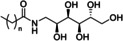	**15** 95 %	**16** 86 %	**17** 86 %	**18** 18 %
**3**	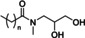	**19** 39 %	**20** 38 %	**21** 34 %	**22** 12 %
**4**	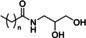	**23** 78 %	**24** 81 %	**25** 83 %	**26** 33 %
**5**	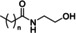	**27** 74 %	**28** 71 %	**29** 77 %	**30** 35 %
**6**	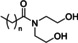	**31** 11 %	**32** 9 %	**33** 7 %	**34** <1 %

[a] Reaction conditions: Carboxylic acid (5 mM), amine (50 mM), AMP (17.1 mM), MgCl_2_ (66.5 mM), Polyphosphate (14.9 mg mL^−1^), CHU (0.27 mol %), CAR*mm*‐A (0.55 mol %), HEPBS buffer (100 mM), 1 % DMSO, 0.5 mL scale, pH 8.5, 37 °C, 250 rpm, 16 h.

To demonstrate the synthetic utility of this biocatalytic method, we scaled up representative examples (**11**, **15** and **25**) to 30 mL in order to isolate the reaction products for analysis. The commercial surfactant MEGA‐8 (**11**, 42 % yield) was obtained using normal‐phase flash chromatography and compound **15** (54 % yield) and ceramide analogue **25** (78 % yield) were successfully obtained after reverse phase flash chromatography (Supporting Information).

Our results suggest that the CAR*mm*‐A‐based amide synthesis is more selective than lipase‐based methods, even though this reaction is a promiscuous activity for both enzyme classes. However, the reaction paths for each enzyme are very different as shown in Figure 3. The acylation reaction catalyzed by lipases is expected to proceed via an acyl‐enzyme intermediate using the classic triad mechanism (Figure 3A).^[29]^ The selectivity of the nucleophile (N vs O) would be expected to be controlled by the enzyme, with lipases naturally evolved to prefer ester substrates. A change in reactivity would involve protein engineering, with some interesting examples on catalytic triad enzymes reported recently.^[23]^

**Figure 3:**
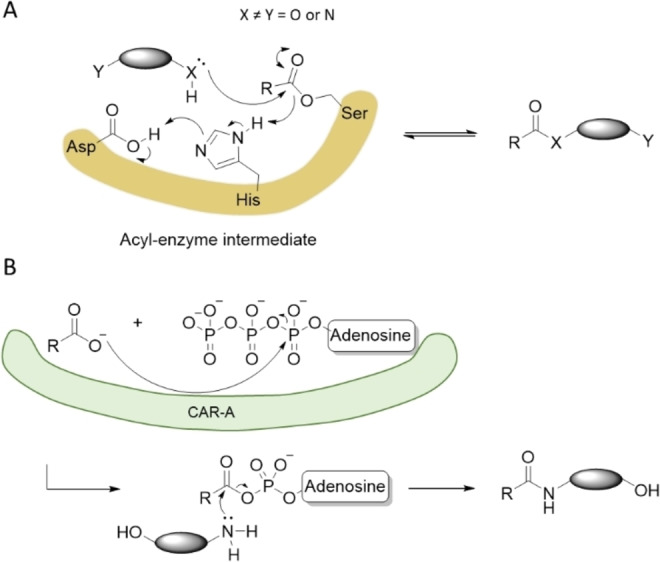
Comparison of the mechanisms of lipase and CAR*mm*‐A catalyzed reactions between fatty acids/esters and amino alcohols. A) Lipase active site residues (in yellow) promoting the reaction via formation of an acyl‐enzyme intermediate. B) Proposed mechanism for CAR*mm*‐A catalysed amidation: active‐site adenylation step to form an acyl adenylate intermediate that preferentially reacts with amines over alcohols.

In contrast, CAR*mm*‐A would be expected to generate a fatty acid‐acyl‐AMP intermediate (figure 3B). The work by Chaiyen et al. shows that this intermediate does not react readily with alcohols: significant amounts of imidazole have to be added to the reaction mixture to be able to observe ester formation, i.e. requiring imidazole as a catalyst.^[20]^ Thus, we suggest that the selectivity is controlled by the intrinsic preference of acyl phosphates in water for amide over ester formation, which is documented in the literature.[^30^, ^31^]

In summary, this study demonstrates the versatility of CAR*mm*‐A for one‐step, selective amide bond formation under aqueous conditions by starting from otherwise challenging, multifunctional substrates. It provides a highly selective alternative to current synthesis methods of MEGAs and related surfactants directly from acid and amines without ester‐forming side reactions observed in lipase‐catalyzed reactions. This amidation method might provide a valuable starting point for further reaction engineering and application in more industrially relevant processes.

## Conflict of interest

The authors declare no conflict of interest.

## Supporting information

As a service to our authors and readers, this journal provides supporting information supplied by the authors. Such materials are peer reviewed and may be re‐organized for online delivery, but are not copy‐edited or typeset. Technical support issues arising from supporting information (other than missing files) should be addressed to the authors.

Supporting Information

## Data Availability

The data that support the findings of this study are available in the Supporting Information of this article.
