# Supplementary material for: One‐Step Biocatalytic Synthesis of Sustainable Surfactants by Selective Amide Bond Formation
Source: Angew Chem Int Ed Engl. 2022 Jun 8;61(30):e202205054. doi: 10.1002/anie.202205054 (PMC9401052; doi:10.1002/anie.202205054)
Supplement: Supplementary file 1 — Supporting Information [file ANIE-61-0-s001.pdf]

Angewandte Chemie
Eine Zeitschrift der Gesellschaft Deutscher Chemiker

Supporting Information

One-Step Biocatalytic Synthesis of Sustainable Surfactants by Selective Amide Bond Formation

*M. Lubberink, W. Finnigan, C. Schnepel, C. R. Baldwin, N. J. Turner, S. L. Flitsch**

Table of Contents

Methods and materials 3
Materials and instrumentation 3
Protein expression and purification 3
Biotransformation procedure 3
Acylation of ^{13}C-decanoic acid 4
^{19}F-NMR 5
Optimisation 8
Analysis of analytical scale biotransformations 12
Preparative scale reactions 25
MEGA-8 (**11**) 25
N-octanoyl glucamine (**15**) 25
N-(2,3-dihydroxypropyl)decanamide (**25**) 26
Gene sequences 27
CAR*mm*Δ647-1175 27
CHU 27
References 28
Compound spectra 29

Methods and materials

Materials and instrumentation

All chemicals and buffers were bought from Sigma Aldrich, Fluorochem or Fischer Scientific. Medium for cell growth was bought from Formedium. All materials relating to molecular biology work were purchased from New England Biolabs (NEB). All NMR spectra were recorded using a Bruker Avance 400 instrument.

HPLC analyses were performed using an Agilent 1260 Infinity II system.

LC/MS analyses were performed using an Agilent 1200 series LC system equipped with a G1379A degasser, a G1312A binary pump, a G1329 autosampler unit, a G1316A temperature-controlled column compartment and a G1315B diode array detector. Compounds were ionized using API-electrospray technique and detected in positive mode on the LCMS System. Drying gas temperature 250 °C at 12 L min-1, and nebulizer pressure at 25 psig.

On both LC/MS and HPLC systems an ACE5 C18 column was used (Dimensions: 250 x 4.6 mm).

For HRMS analyses an Agilent 1200 series LC system was used, coupled to an Agilent 6520 QTOF mass spectrometer, ESI positive mode. The data was analysed using Agilent MassHunter software.

Protein expression and purification

CAR*mm*-A and CHU genes, plasmids and expression strains (*E. coli* BL21 (DE3)) were prepared using previously described methods.[1]

For protein expression, autoclaved baffled flasks containing 700 ml auto-induction medium containing the appropriate antibiotic, were inoculated with *E. coli* BL21 (DE3) cells and were grown at 30°C for 72 hours. Cells were harvested by centrifugation and the cell pellet was stored in zip-lock bags at -80°C.

To lyse the cells for purification, the cell pellet was resuspended in Equilibration buffer (50 mM Tris.HCl pH 8, 200 mM NaCl). The cells were then sonicated 20s on/20s off 25 times. The lysis mixture was subsequently centrifuged, the supernatant collected and the pellet discarded. The supernatant was then mixed with Ni-NTA agarose and left shaking at 4°C for 30 minutes. This mixture was then poured into a gravity column and was washed with Wash Buffer (10 mM imidazole, 50 mM Tris.HCl pH 8, 200 mM NaCl). The protein was then eluted using Elution Buffer (200 mM imidazole, 50 mM Tris.HCl pH 8, 200 mM NaCl). The eluted protein was concentrated using Vivaspin centrifugal concentrators (30.000 MWCO, Sartorius) and then desalted using PD-10 columns (GE Healthcare) following the respective protocols. Purity was checked using SDS-PAGE (staining with Instant Blue (Expedeon)) and concentration was determined by measuring absorbance at 280 nm using Nanodrop (Thermo Fisher).

For the production of cell-free lysates, the frozen cell pellets were resuspended in reaction buffer (100 mM HEPBS pH 8.5), then sonicated and subsequently centrifuged as described above. The supernatant protein concentration was measured, aliquoted and stored at -20°C.

Biotransformation procedure

In an example CAR*mm*-A biotransformation, 5 mM of the carboxylic acid substrate (from a 0.5M stock in DMSO), 50 mM of amine (from a 0.25 M stock in buffer, adjusted to pH 8.5), 17.1 mM ATP (from a 0.1 M stock in buffer, adjusted to pH 8.5), 66.5 mM $MgCl_2$ and CARmm-A (1 mg/mL) were added to HEPBS buffer (100 mM, pH 8.5) to a total volume of 0.5 mL in a 1.5 mL Eppendorf tube. The reaction was placed in a 37°C incubator for 16 hours shaking at 250 rpm.

The reaction was stopped by adding an equal volume of MeOH and shaking the mixture. This mixture was centrifuged and the supernatant was filtered and added to an HPLC vial for analysis.

Acylation of ^{13}C-decanoic acid

To investigate the reaction selectivity of amidation versus esterification, ^{13}C-labelled decanoic acid was reacted with amino sugar **1** (Figure S1).

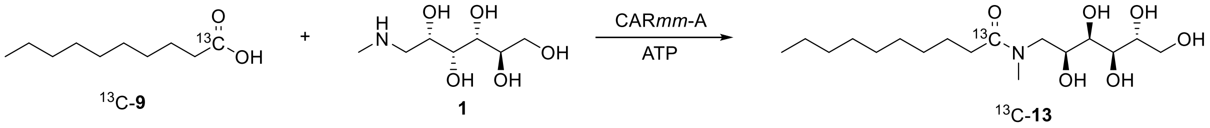

Figure S1: Reaction scheme for the CAR*mm*-A catalysed reaction between ^{13}C-labelled **9** and **1** to synthesise ^{13}C-labelled **13**. Reaction conditions: Carboxylic acid (10 mM), 1 (175 mM), ATP (50 mM), MgCl2 (50 mM), CAR*mm*-A (14 μM), HEPBS buffer (100 mM), 5% DMSO, 0.5 mL scale, pH 8.5, 37 °C, 250 rpm, 16 h.

The crude biotransformation was mixed 1:1 with MeOD and analysed using ^{13}C-NMR. For a negative control, a reaction without adding the enzyme catalyst was included, as well as a commercial standard of MEGA-10. The carbonyl regions of the ^{13}C-NMR spectra of these samples are shown in Figure S2.

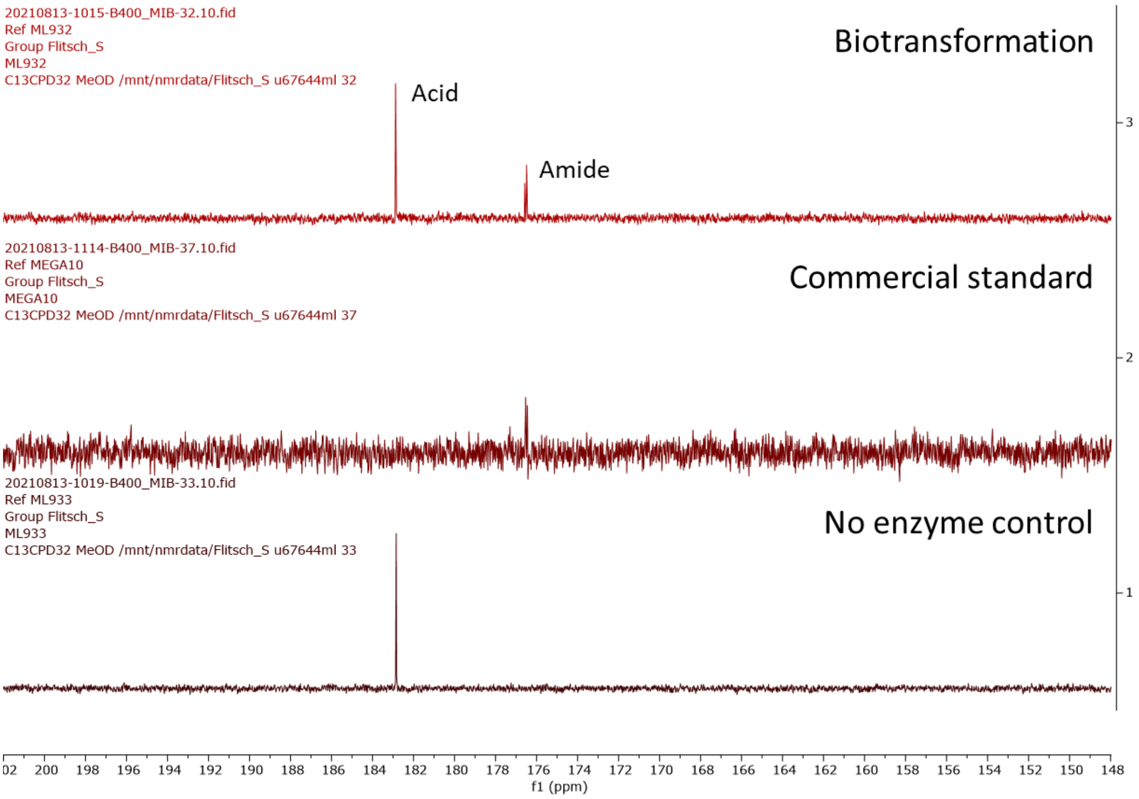

Figure S2: ^{13}C-NMR (MeOD, 101 MHz) spectra of the CAR*mm*-A catalysed biotransformation between ^{13}C-labelled decanoic acid and **1** (top), a commercially bought standard of MEGA-10 (middle) and the no enzyme control (bottom), zoomed in to the carbonyl region of the spectrum (~150-190 ppm).

As the carbonyl region of the ^{13}C-NMR spectrum does not show any peaks other than the acid and amide peaks, it suggests the reaction is selective towards a single product without any unwanted ester byproducts.

^{19}F-NMR

To investigate the activity of amino sugars, initial CAR*mm*-A catalysed biotransformations using 3-fluoro cinnamic acid and amino sugar **1** were performed and analysed by ^{19}F-NMR (using previously described methods[1], figure S3).

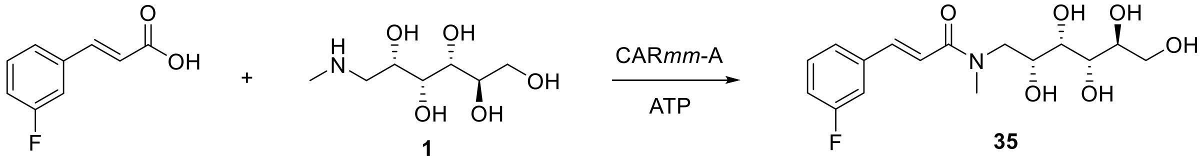

Figure S3: Reaction conditions: Carboxylic acid (5 mM), 1 (175 mM), ATP (50 mM), MgCl2 (50 mM), CAR*mm*-A (1 mg/mL), HEPBS buffer (100 mM), 5% DMSO, 0.5 mL scale, pH 8.5, 37 °C, 250 rpm, 16 h.

Figure S4 shows the crude biotransformation (top) and the same biotransformation when spiked with the 3-fluoro cinnamic acid substrate (bottom). This indicated that the substrate in the biotransformation has been completely converted to product.

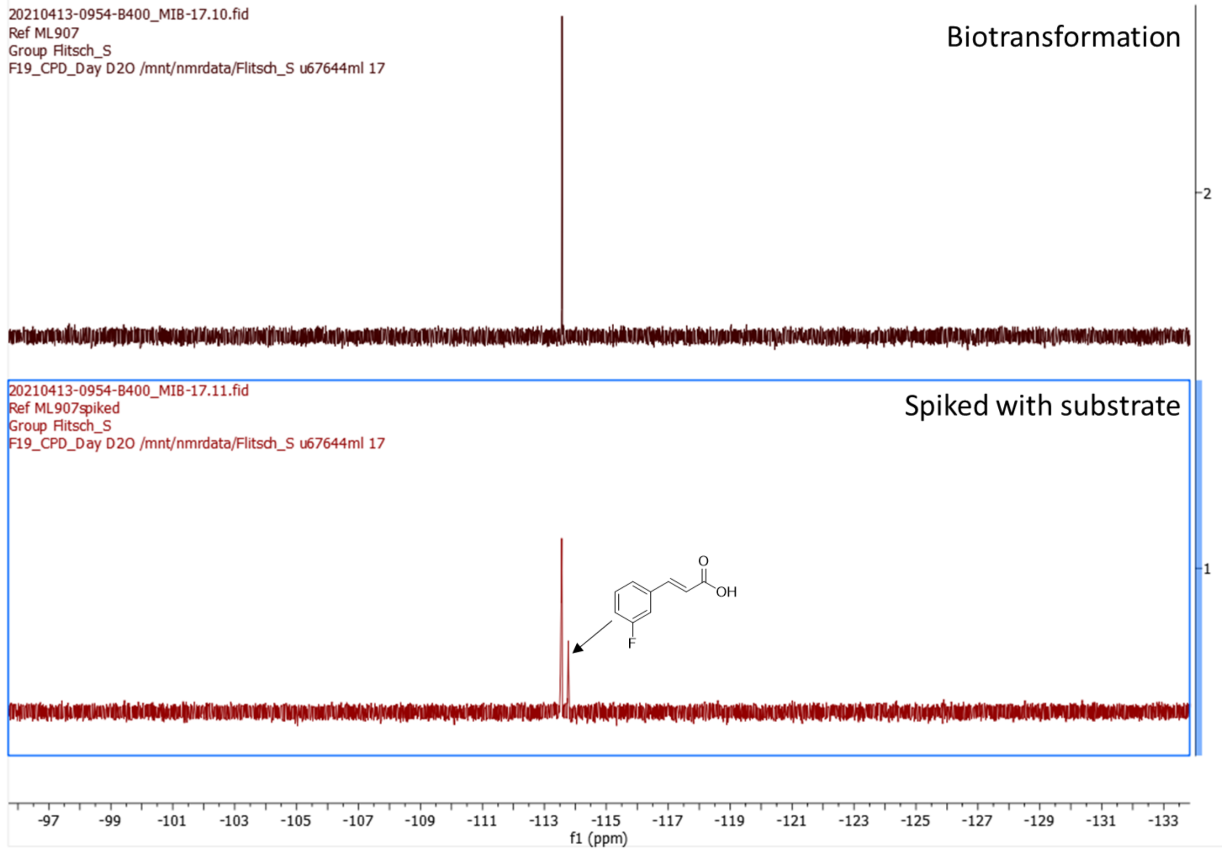

Figure S4: ^{19}F-NMR spectra of the crude biotransformation between 3-fluoro cinnamic acid and amino sugar **1** (top) and the same biotransformation spiked with 3-fluoro cinnamic acid (bottom).

To identify the reaction product in this reaction, it was additionally analysed by LC-MS (Figure S5). It was found again that the substrate had been fully converted to the product. The product showed m/z values at 344, 366 and 382 which correspond to the mass of amide **35** ([M+H], [M+Na] and [M+K] respectively).

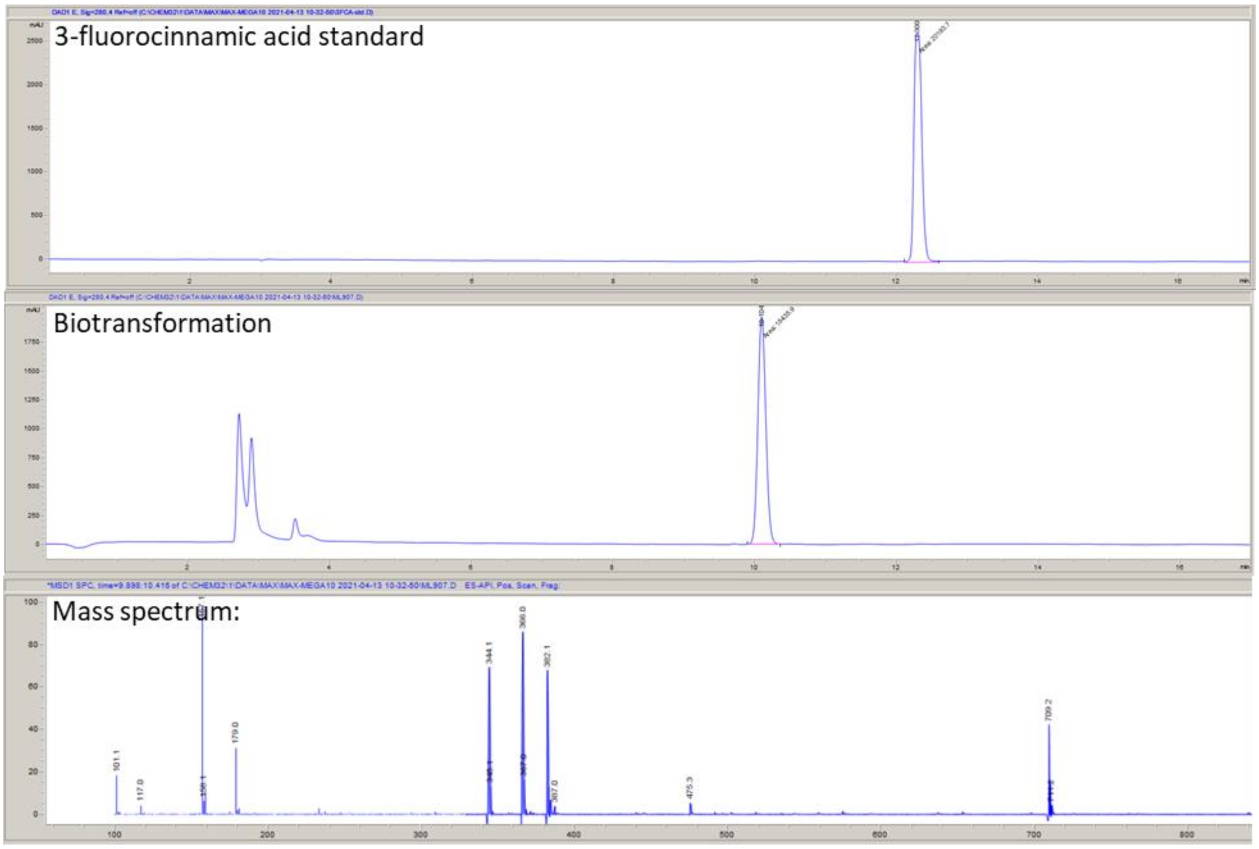

Figure S5: LC chromatogram of the 3-fluoro cinnamic acid substrate at 280nm (top), LC chromatogram of the crude CAR*mm*-A catalysed reaction between 3-fluoro cinnamic acid and **1** at 280 nm (middle), and the mass spectrum of the product peak from the middle LC chromatogram showing m/z values at 344, 366 and 382 (bottom).

We also investigated wether sorbitol (a poly-alchohol derivative of glucose) would lead to ester formation when used as a nucleophile using the optimized reaction conditions and 3-fluoro cinnamic acid (Figure S6). We observed that the ^{19}F-NMR spectra for the sorbitol experiment was identical to the experiment that contained no nucleophile. A very small new peak appeared in these experiments which is the small amount of acyl adenylate that is present in solution. This peak did not show in the no enzyme control experiment as expected. Therefore we concluded that no ester formation occurs when using sorbitol as a nucleophile. Furthermore, a positive control reaction between 3-fluoro cinnamic acid and **1** was performed showing full conversion to the amide product.

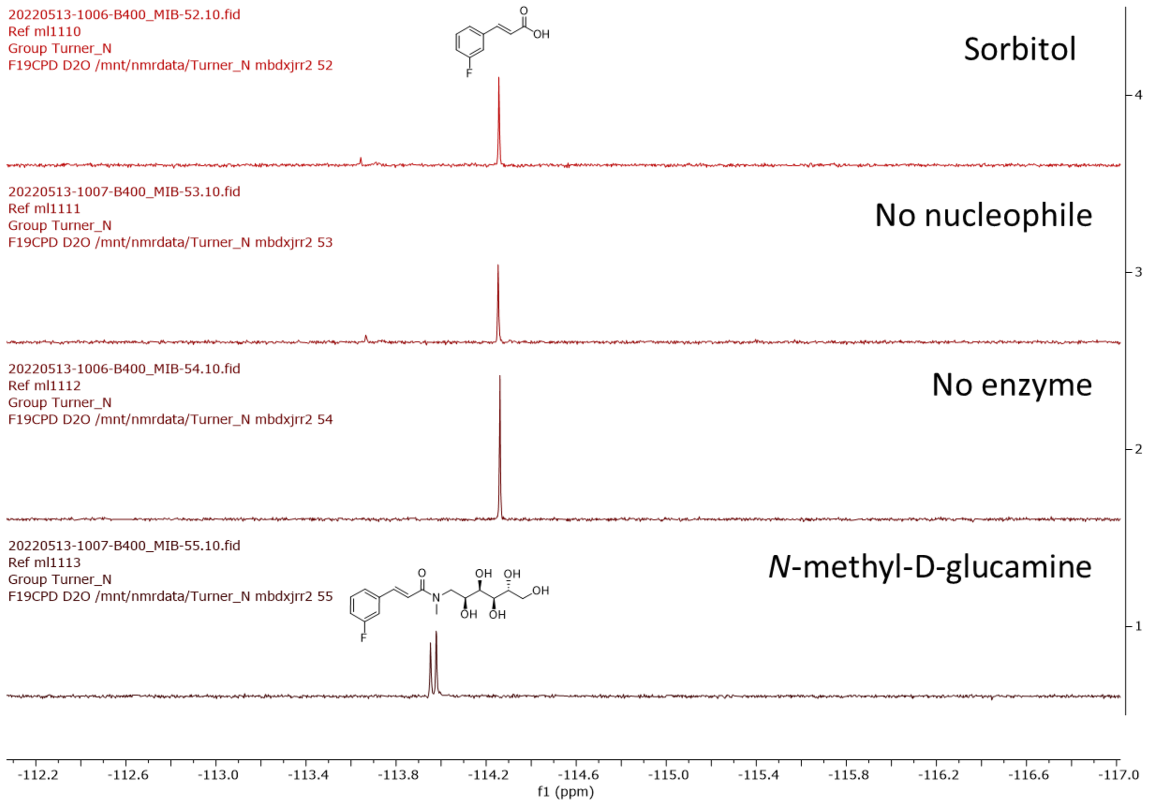

Figure S6: ^{19}F-NMR spectra of the crude biotransformation between 3-fluoro cinnamic acid and sorbitol (top), with no nucleophile (2nd row), with no enzyme (3rd row), and a positive control using *N*-methyl-D-glucamine as a nucleophile (bottom). Reaction conditions: Carboxylic acid (5 mM), nucleophile (50 mM), AMP (17.1 mM), MgCl2 (66.5 mM), Polyphosphate (14.9 mg/ml), CHU (13 µM), CAR-A (28 µM), HEPBS buffer (100 mM), 1% DMSO, 0.5 mL scale, pH 8.5, 30 °C, 250 rpm, 16 h.

Optimisation

Initial reaction test for reacting **7** with **1** resulting in MEGA-8 (**11**) was performed using previously reported conditions using an excess of amine and ATP.[1] The conversion was determined by RP-HPLC at a wavelength of 210 nm, using a commercial standard as a reference for a calibration curve (Figure S7 and S8). The calculated conversion of this reaction was >99%.

Conc. (mM)	Signal (mAU)
0.625	687
1.25	1366
2.5	2708
5	5684
10	11350

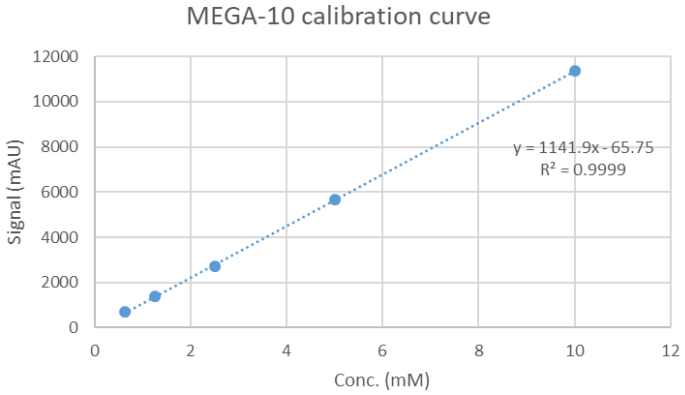

Figure S7: Signal values for the different concentrations of the dilution series of MEGA-10 (left) and the values plotted in a calibration curve (right).

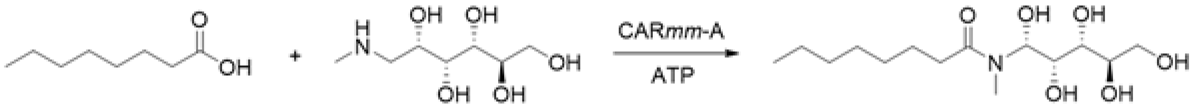

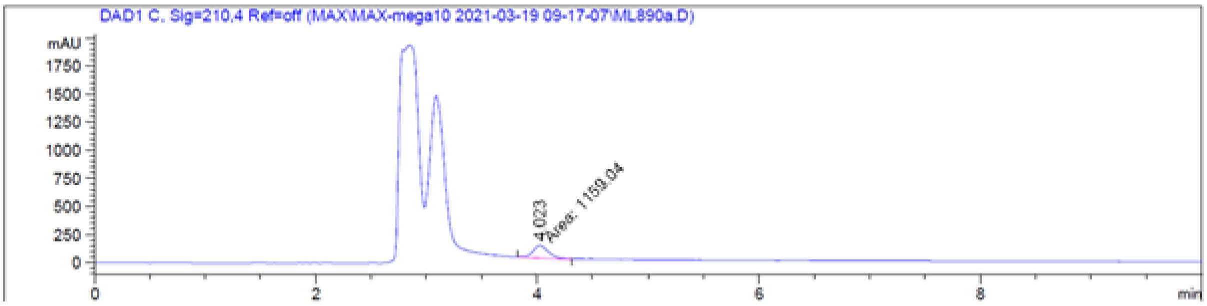

Figure S8: Reaction scheme for the CAR*mm*-A catalysed reaction between octanoic acid and **1** to synthesise **2**. HPLC chromatograms showing the reaction using 1 mM octanoic acid, with the product peak integrated showing it's mAU value. Reaction conditions: Octanoic acid **7** (1 mM), Amine **1** (175 mM), ATP (50 mM), MgCl2 (50 mM), CAR*mm*-A (1 mg/mL), HEPBS buffer (100 mM), 5% DMSO, 0.5 mL scale, pH 8.5, 37 °C, 250 rpm, 16 h.

For reaction optimisation we used the reaction between **9** and **1** resulting in MEGA-10 (**13**), using the CHU enzyme to regenerate ATP from AMP and polyphosphate as a model reaction (figure S9).

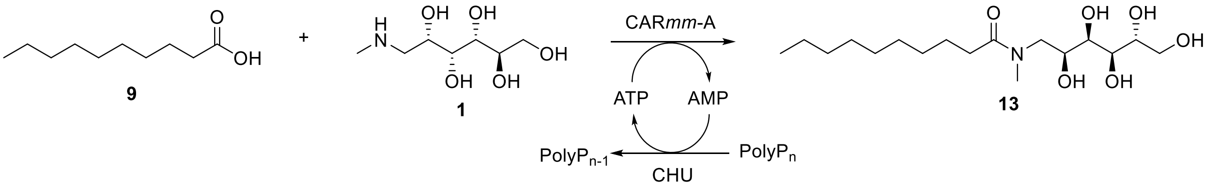

Figure S9: Reaction scheme for the CAR*mm*-A catalysed reaction between **9** and **1** to synthesise **13**, using the CHU enzyme to regenerate ATP from AMP and polyphosphate.

Using previously reported reaction conditions we investigated the effect of amine concentration on the conversion of substrates to **13** (Figure S10).

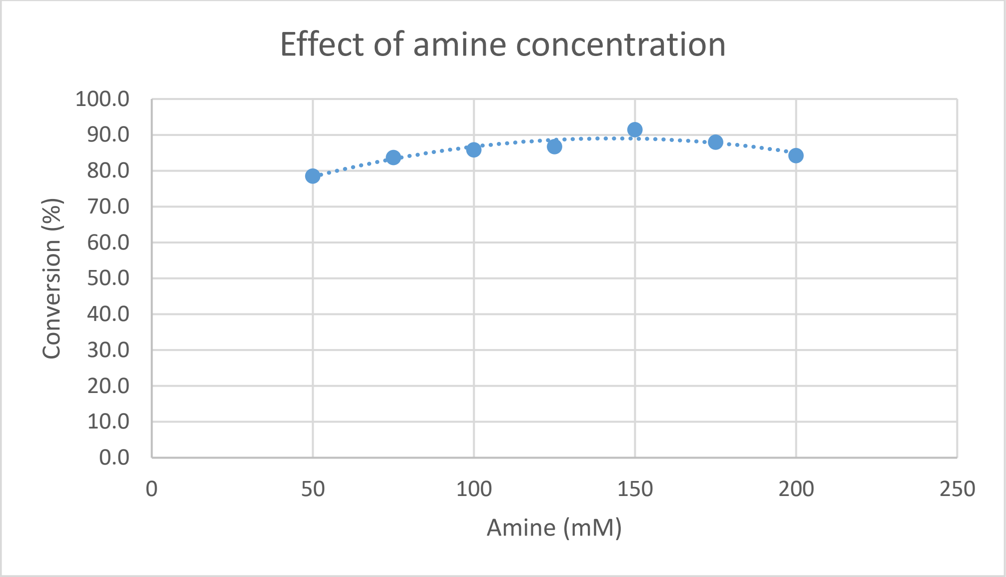

Figure S10: Effect of amine concentration on conversion to **13**. Reaction conditions: Carboxylic acid (5 mM), amine (range), AMP (10 mM), MgCl2 (50 mM), Polyphosphate (100 mg/ml), CHU (13 µM), CAR-A (28 µM), HEPBS buffer (100 mM), 5% DMSO, 0.5 mL scale, pH 8.5, 37 °C, 250 rpm, 16 h.

We performed design of experiments using the software JMP®, (Version 16 Pro. SAS Institute Inc., Cary, NC, 1989–2022) to optimize and better understand the CHU system. We constructed an empirical model for the effect of polyphosphate, AMP and Mg^{2+} concentrations on conversion using data from a set of biotransformation conditions generated by the software (Figure S11). Using the maximize desirability option in the prediction profiler, it was found that the optimum reaction conditions were 17.1 mM AMP, 66.5 mM MgCl2, and 14.9 mg/ml polyphosphate.

Run	$MgCl_2$ (mM)	PolyP (mg/ml)	AMP (mM)	Conversion (%)
1	5	3	2.5	27
2	52.5	30	25	58
3	5	3	25	31
4	100	3	25	41
5	52.5	16.5	13.75	84
6	100	16.5	25	68
7	52.5	16.5	13.75	84
8	100	16.5	2.5	26
9	5	30	13.75	0
10	100	30	13.75	64
11	52.5	3	13.75	69
12	52.5	16.5	13.75	82
13	52.5	30	2.5	29
14	5	20.955	25	0
15	100	3	2.5	24
16	5	19.875	2.5	0

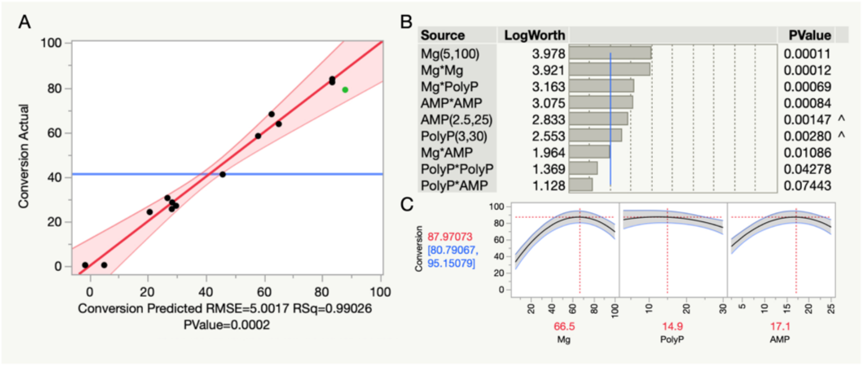

Figure S11: Conversions of the set of biotransformations generated by the JMP software (table) and an empirical model of the reaction. (A) The actual conversion plotted against the model prediction R^2 = 0.99. (B) Pareto plots of model factor significance. (C) Snapshot of the prediction profiler set to maximum desirability, showing the optimum concentrations of Mg^{2+}, PolyP and AMP as predicted by the model.

Using these optimum conditions, we further explored the effect of amine concentration (Figure S12).

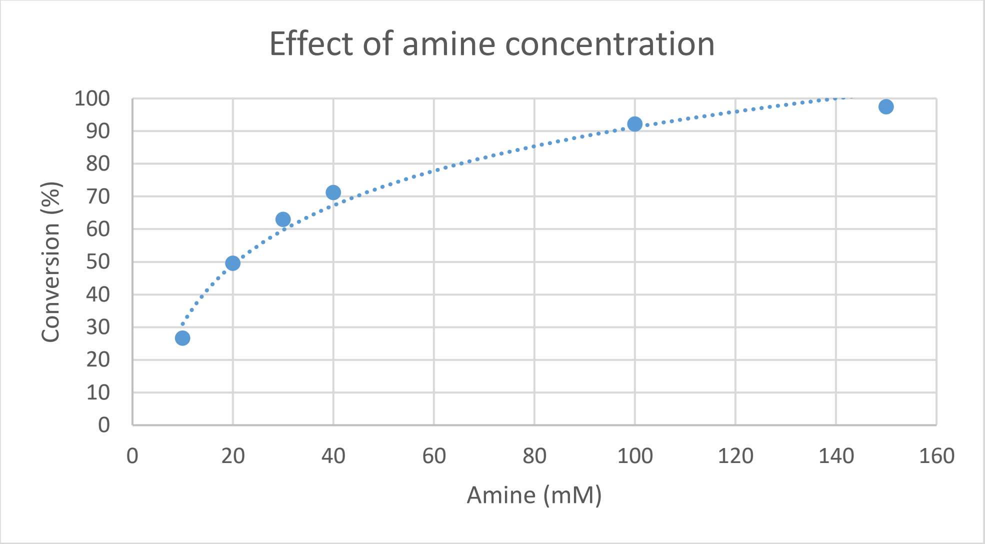

Figure S12: Effect of amine concentration on conversion to **13**. Reaction conditions: Carboxylic acid (5 mM), amine (range), AMP (17.1 mM), MgCl2 (66.5 mM), Polyphosphate (14.9 mg/ml), CHU (13 µM), CAR-A (28 µM), HEPBS buffer (100 mM), 5% DMSO, 0.5 mL scale, pH 8.5, 37 °C, 250 rpm, 16 h.

We then performed another round of design of experiments to gain more insight into the effects of acid, amine and CAR concentration on the conversion and the analytical yield. (Figure S13)

Run	Acid (mM)	Amine (mM)	CAR (mg/mL)	Analytical Yield (mM)	Conv. (%)
1	5	60	2	4.49	90
2	8	80	0.5	3.08	39
3	2	60	1.25	1.88	94
4	2	80	0.5	1.97	98
5	5	60	0.5	3.83	77
6	2	40	2	1.82	91
7	5	80	1.25	4.55	91
8	5	40	1.25	3.53	71
9	2	40	0.5	1.73	86
10	8	80	2	4.13	52
11	8	60	1.25	3.57	45
12	5	60	1.25	4.39	88
13	5	60	1.25	4.33	87
14	8	40	2	3.77	47
15	2	80	2	2.11	105
16	8	40	0.5	3.05	38

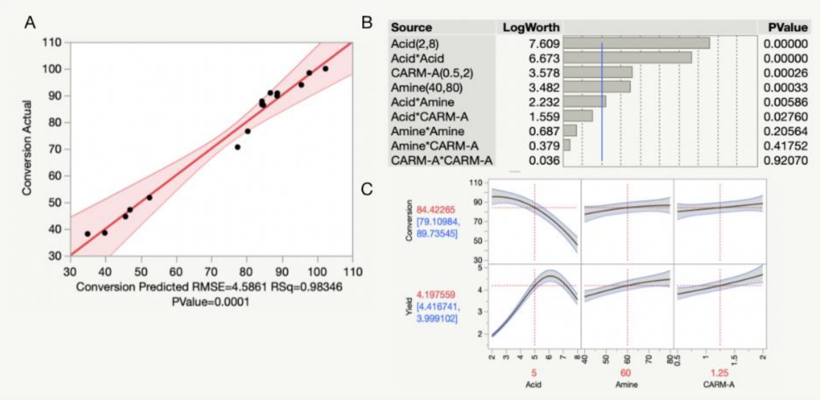

Figure S13: Conversions of the set of biotransformations generated by the JMP software (table) and an empirical model of the reaction. (A) The actual conversion plotted against the model prediction R^2 = 0.98. (B) Pareto plots of model factor significance. (C) Snapshot of the prediction profiler

The effects of co-solvent concentration on the reaction conversion were investigated (Figure S14).

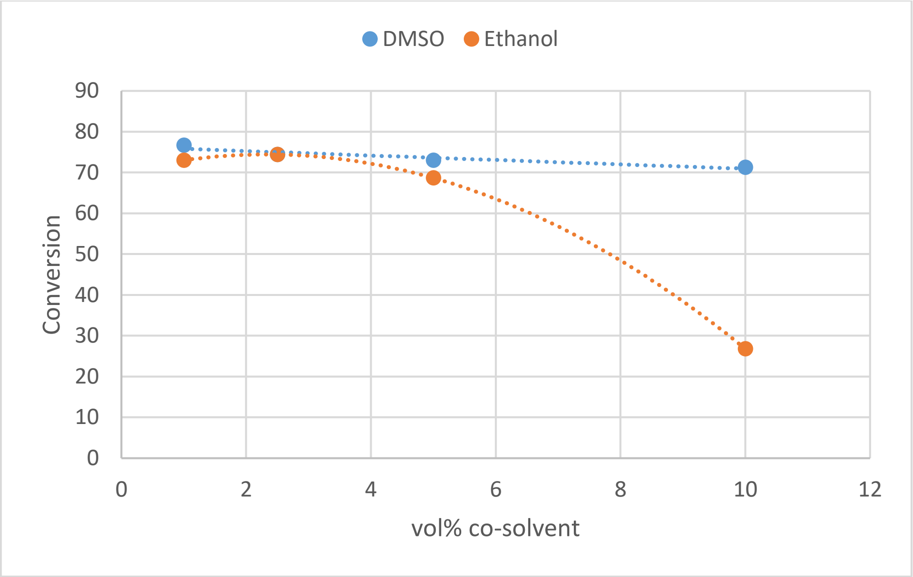

Figure S14: Effect of amine concentration on conversion to **13**. Reaction conditions: Carboxylic acid (5 mM), amine (50 mM), AMP (17.1 mM), MgCl2 (66.5 mM), Polyphosphate (14.9 mg/ml), CHU (13 µM), CAR-A (28 µM), HEPBS buffer (100 mM), co-solvent, 0.5 mL scale, pH 8.5, 37 °C, 250 rpm, 16 h.

Analysis of analytical scale biotransformations

Reactions shown in Table 1 were stopped after 16 hours by adding methanol in a 1:1 ratio, the mixture was centrifuged and the supernatant was used for reversed-phase HPLC and LC/MS analysis.

Method on both LC/MS and HPLC: 10 minute isocratic method of 90:10, 80:20, or 70:30 MeOH (+0.1% TFA)/H_2O (+0.1% TFA).

To calculate conversions, a calibration curve was made using a dilution series of a commercially bought standard of MEGA-10 (**13**) using the concentration range 0.625 mM, 1.25 mM, 2.5 mM, 5 mM and 10 mM. These samples were run using the HPLC conditions described above, detecting the amide at 210 nm, taking the mAU value of the peak of the standard at a retention time of approximately 4.8 min.

The signal values of the dilution series are shown in the table in figure S15 and were plotted in a calibration curve shown in the same figure. The equation of the trend line was then used to calculate the conversions of the *N,N*-di-substituted amides in Table 1 (**11-14**, **19-22**, and **31-34**).

Conc. (mM)	Signal (mAU)
0.625	454
1.25	918
2.5	1831
5	4051
10	8178

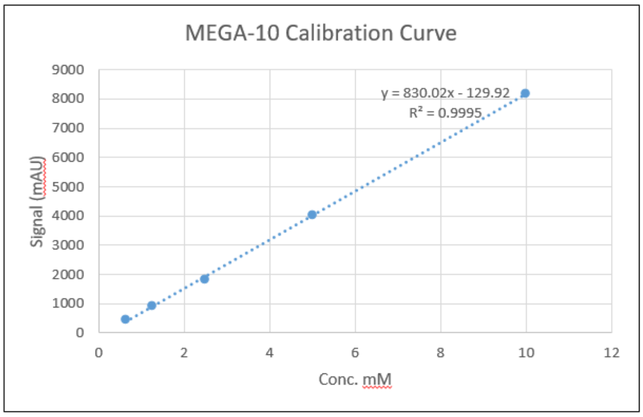

Figure S15: Signal values for the different concentrations of the dilution series of MEGA-10 (left) and the values plotted in a calibration curve (right).

A second calibration curve was made using the same dilution series of a commercially bought standard of lauramide MEA (**30**) (Figure S16). The equation of this trend line was used to calculate the conversions of the mono-*N*-substituted amides in Table 1 (**15-18** and **23-30**).

Conc. (mM)	Signal (mAU)
0.625	116
1.25	246
2.5	490
5	962
10	1925

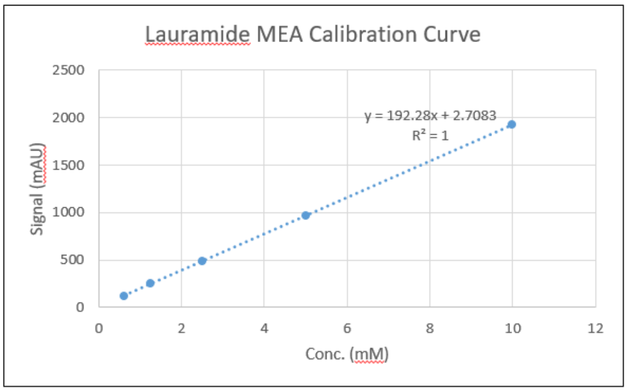

Figure S16: Signal values for the different concentrations of the dilution series of lauramide MEA (left) and the values plotted in a calibration curve (right).

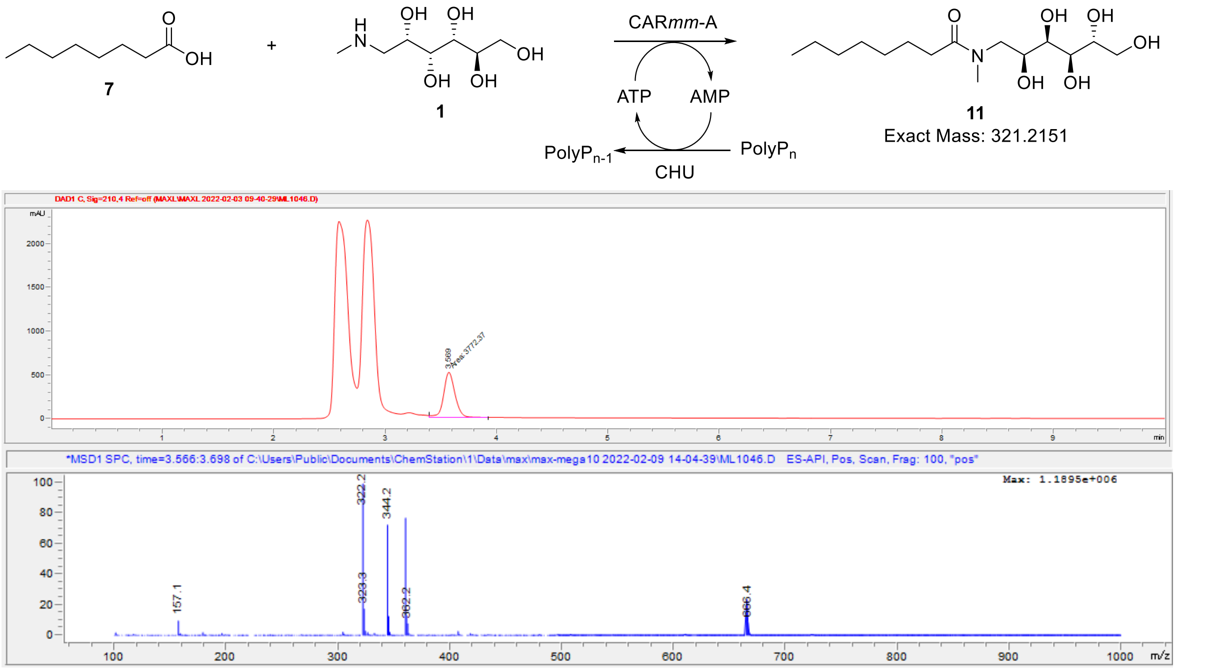

Figure S17: Reaction scheme for the CAR*mm*-A catalysed reaction between **7** and **1** to synthesise **11** (top), LC chromatogram of biotransformation with product peak integrated (middle), and mass spectrum in positive mode of the product peak showing [M+H] = 322, [M+Na] = 344 and [M+K] = 362.

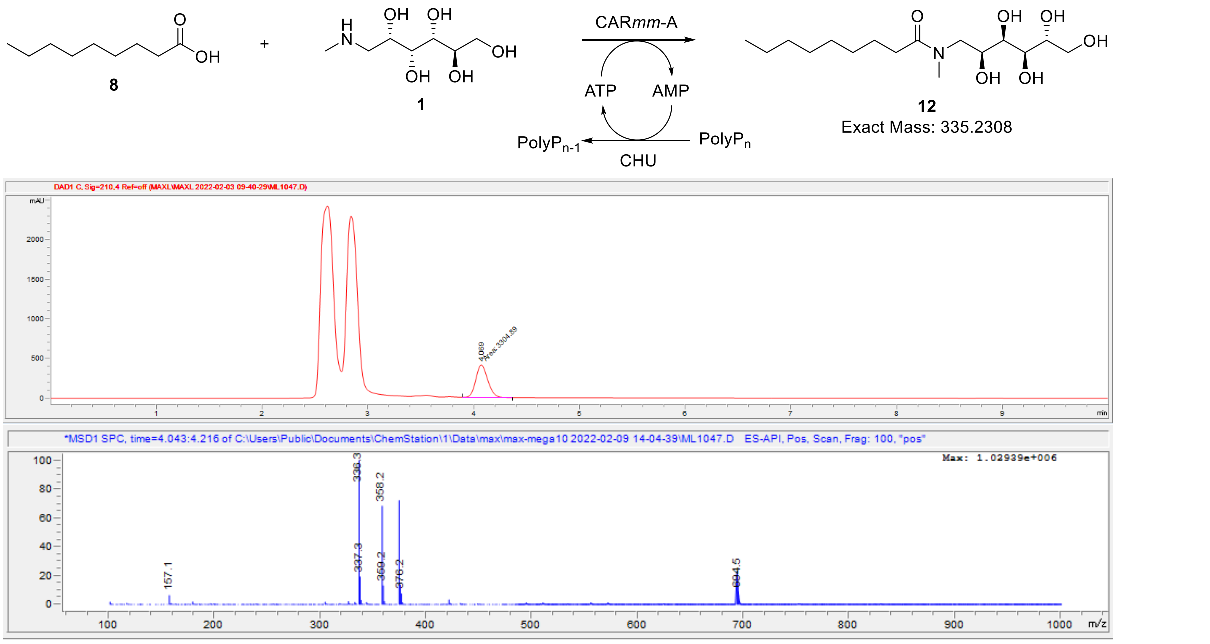

Figure S18: Reaction scheme for the CAR*mm*-A catalysed reaction between **8** and **1** to synthesise **12** (top), LC chromatogram of biotransformation with product peak integrated (middle), and mass spectrum in positive mode of the product peak showing [M+H] = 336, [M+Na] = 358 and [M+K] = 376.

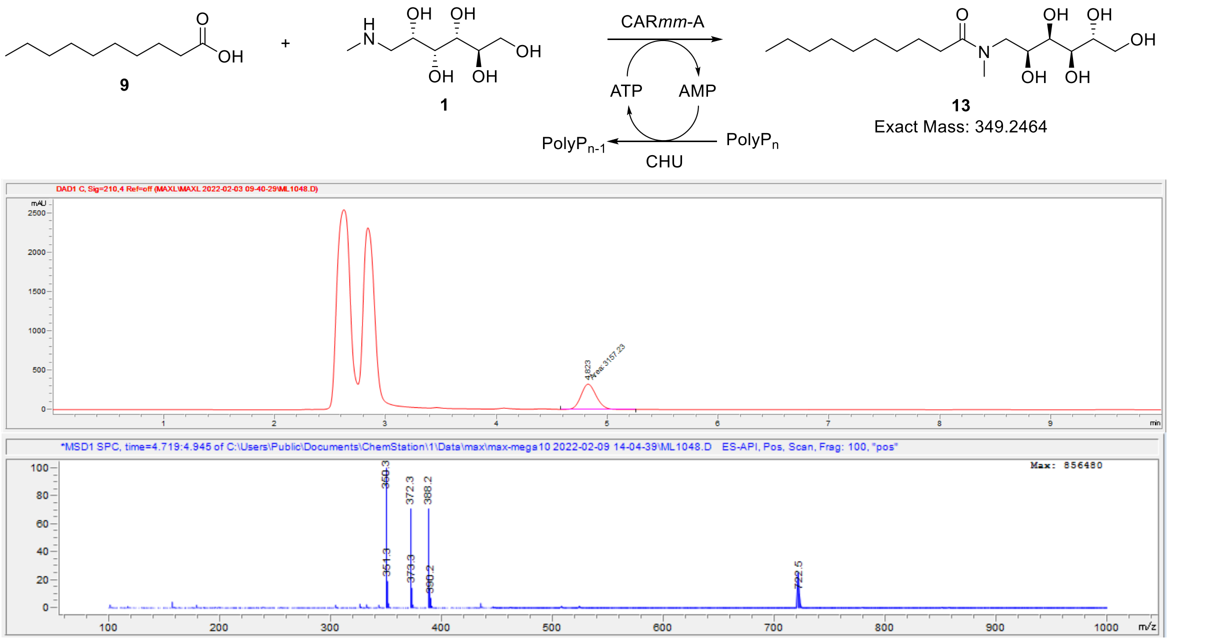

Figure S19: Reaction scheme for the CAR*mm*-A catalysed reaction between **9** and **1** to synthesise **13** (top), LC chromatogram of biotransformation with product peak integrated (middle), and mass spectrum in positive mode of the product peak showing [M+H] = 350, [M+Na] = 372 and [M+K] = 388.

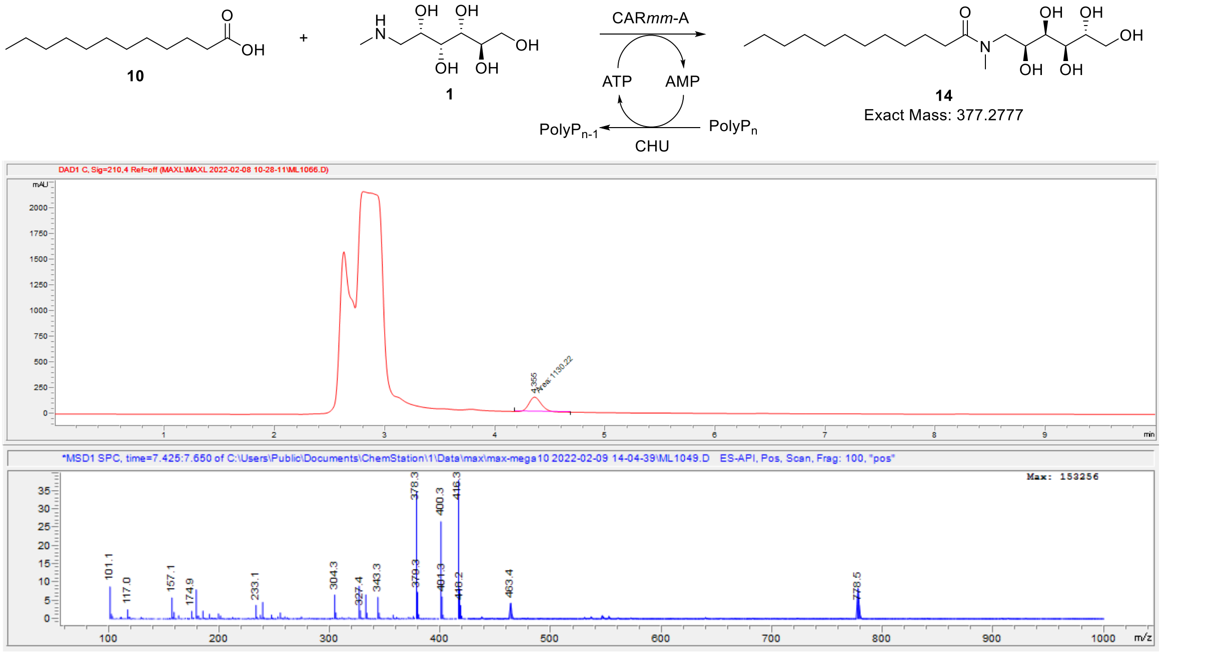

Figure S20: Reaction scheme for the CAR*mm*-A catalysed reaction between **10** and **1** to synthesise **14** (top), LC chromatogram of biotransformation with product peak integrated (middle), and mass spectrum in positive mode of the product peak showing [M+H] = 378, [M+Na] = 400 and [M+K] = 416.

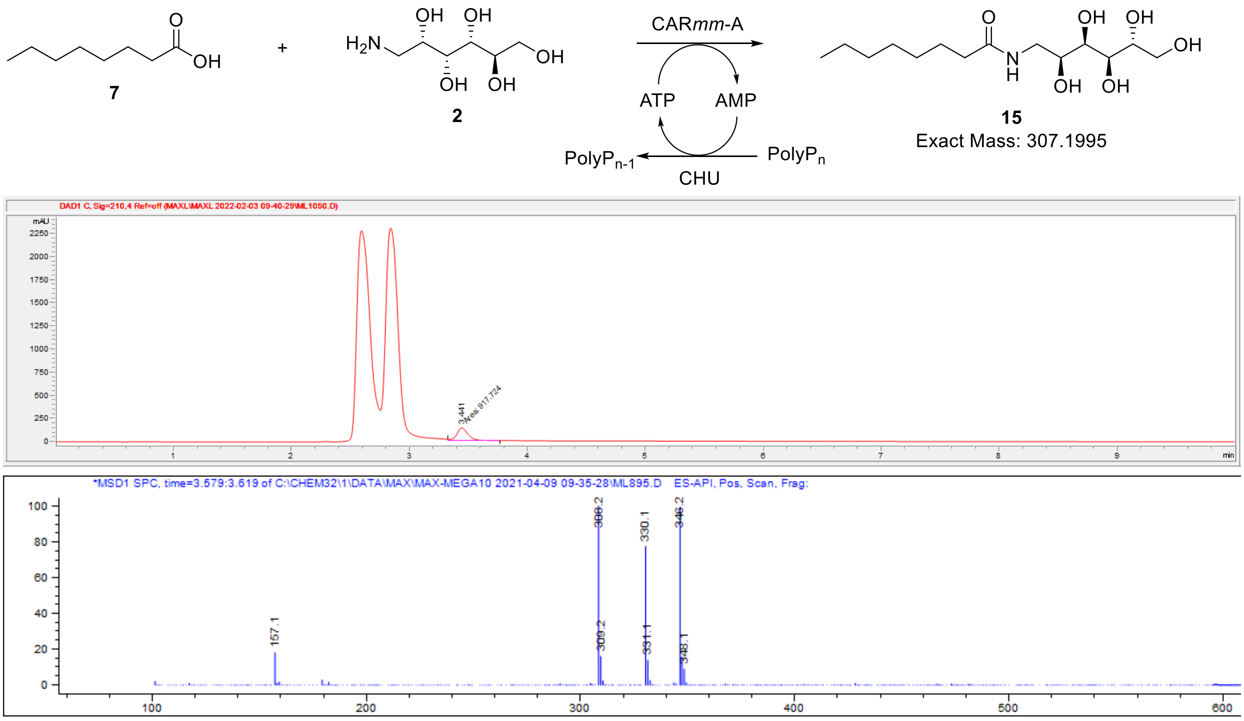

Figure S21: Reaction scheme for the CAR*mm*-A catalysed reaction between **7** and **2** to synthesise **15** (top), LC chromatogram of biotransformation with product peak integrated (middle), and mass spectrum in positive mode of the product peak showing [M+H] = 308, [M+Na] = 330 and [M+K] = 346.

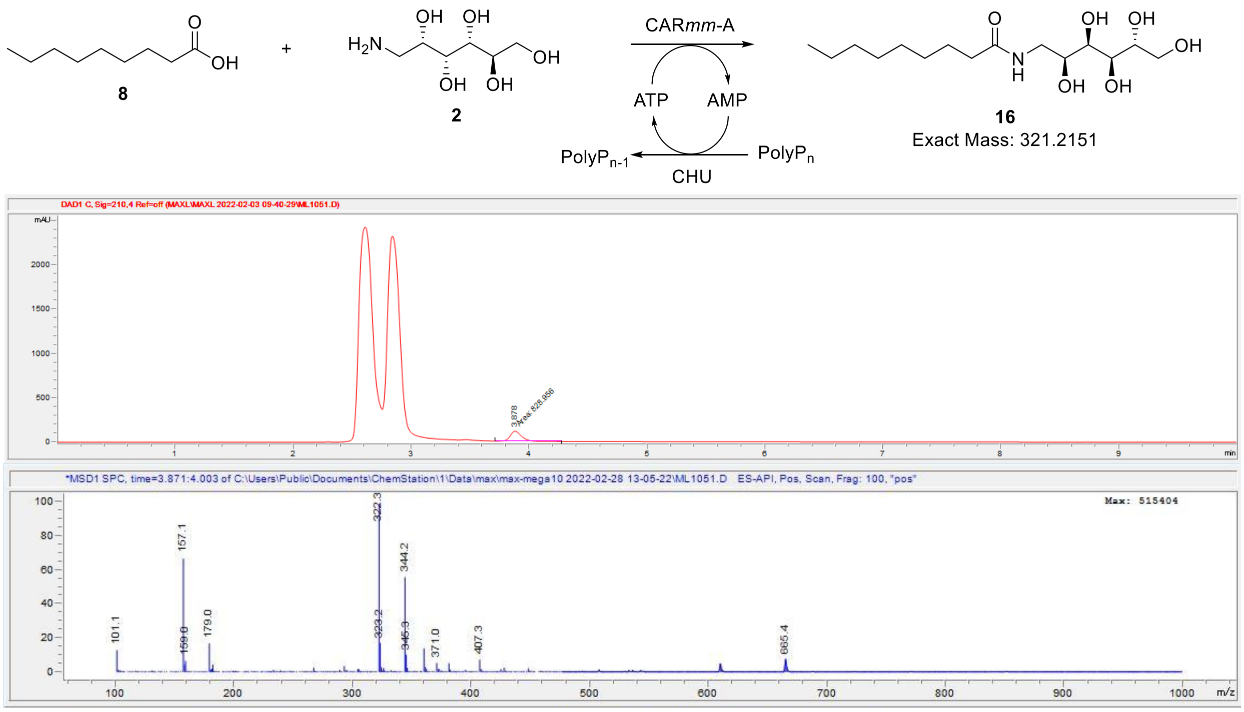

Figure S22: Reaction scheme for the CAR*mm*-A catalysed reaction between **8** and **2** to synthesise **16** (top), LC chromatogram of biotransformation with product peak integrated (middle), and mass spectrum in positive mode of the product peak showing [M+H] = 322 and [M+Na] = 344.

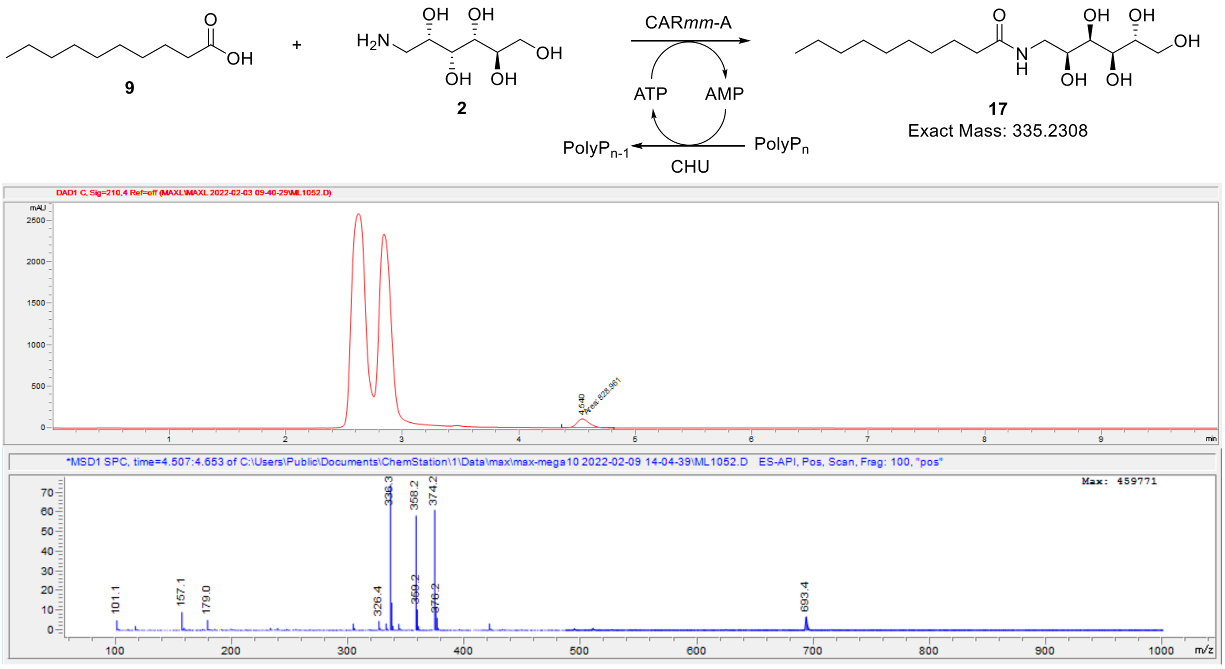

Figure S23: Reaction scheme for the CAR*mm*-A catalysed reaction between **9** and **2** to synthesise **17** (top), LC chromatogram of biotransformation with product peak integrated (middle), and mass spectrum in positive mode of the product peak showing [M+H] = 336, [M+Na] = 358 and [M+K] = 374.

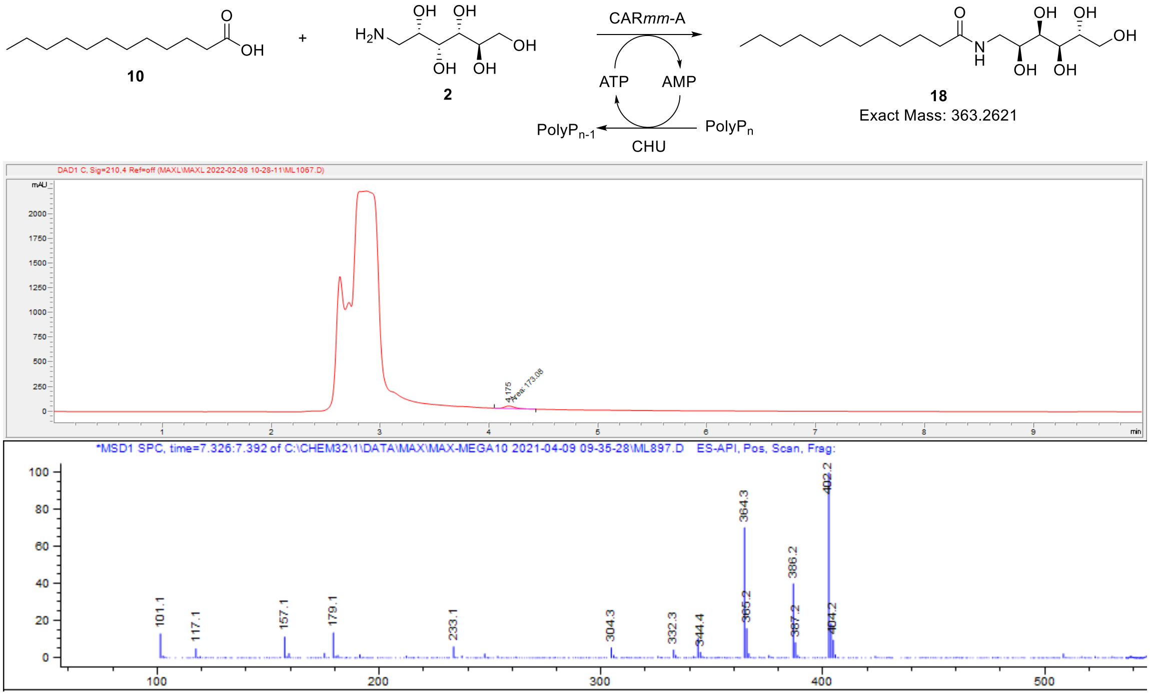

Figure S24: Reaction scheme for the CAR*mm*-A catalysed reaction between **10** and **2** to synthesise **18** (top), LC chromatogram of biotransformation with product peak integrated (middle), and mass spectrum in positive mode of the product peak showing [M+H] = 364, [M+Na] = 386 and [M+K] = 402.

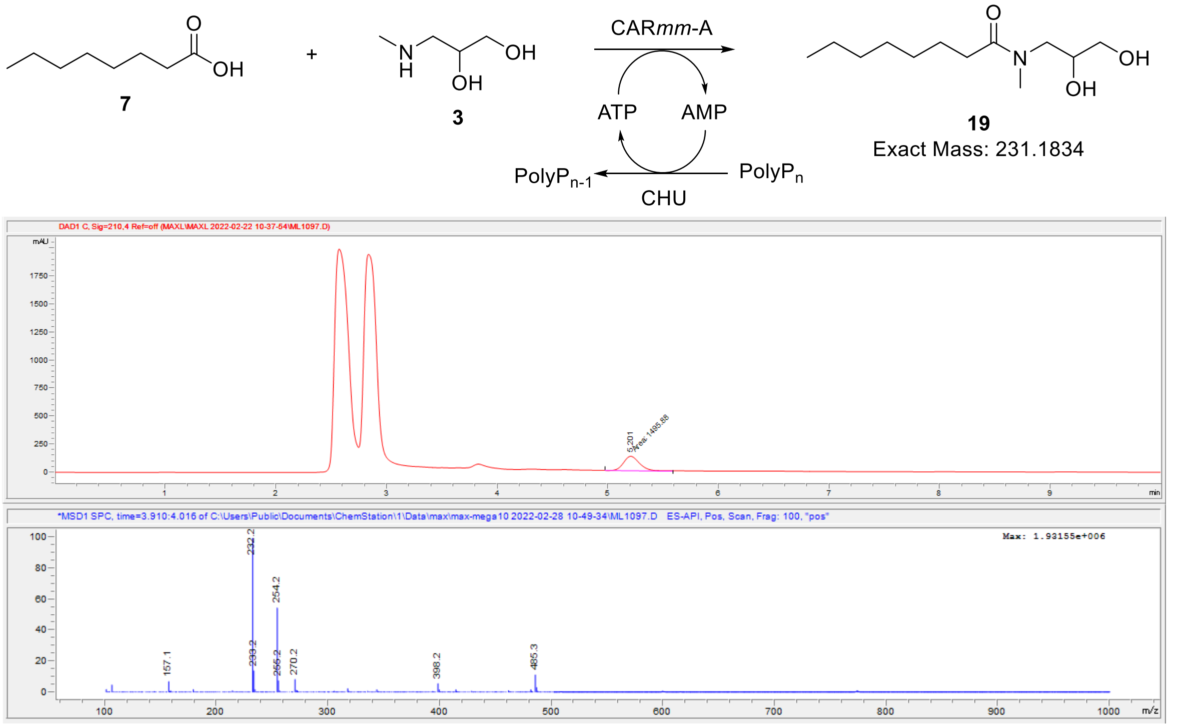

Figure S25: Reaction scheme for the CAR*mm*-A catalysed reaction between **7** and **3** to synthesise **19** (top), LC chromatogram of biotransformation with product peak integrated (middle), and mass spectrum in positive mode of the product peak showing [M+H] = 232, [M+Na] = 254 and [M+K] = 270.

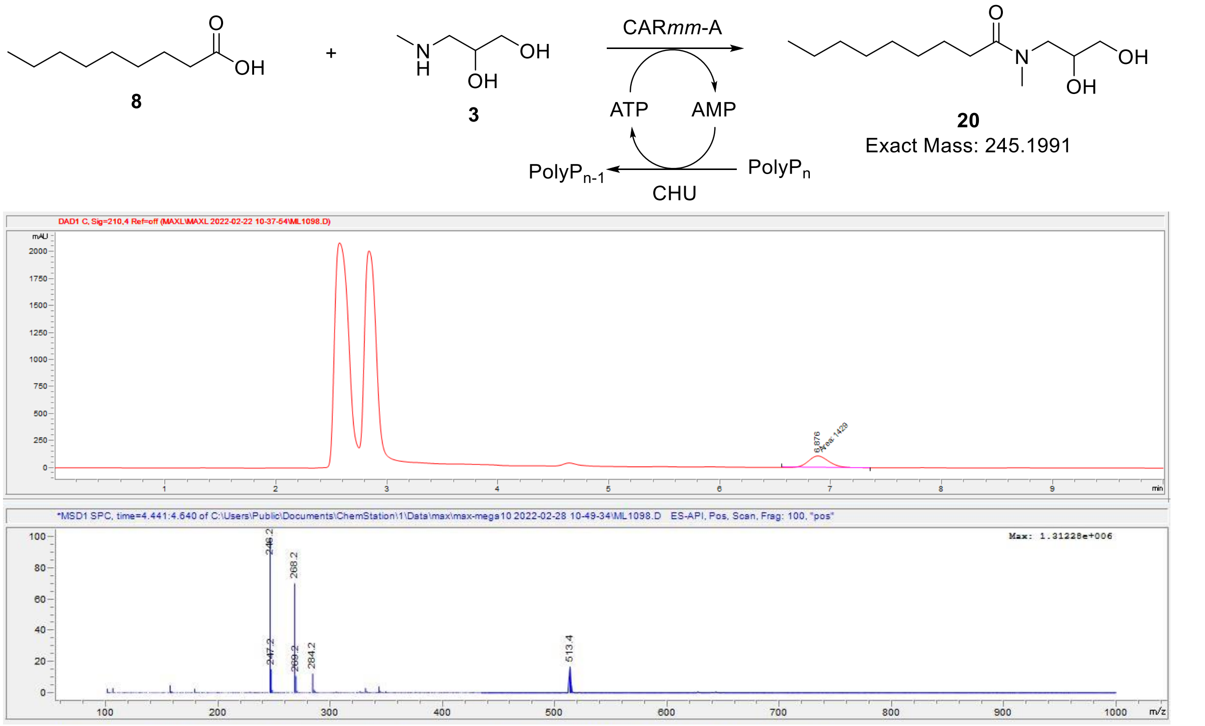

Figure S26: Reaction scheme for the CAR*mm*-A catalysed reaction between **8** and **3** to synthesise **20** (top), LC chromatogram of biotransformation with product peak integrated (middle), and mass spectrum in positive mode of the product peak showing [M+H] = 246, [M+Na] = 268 and [M+K] = 284.

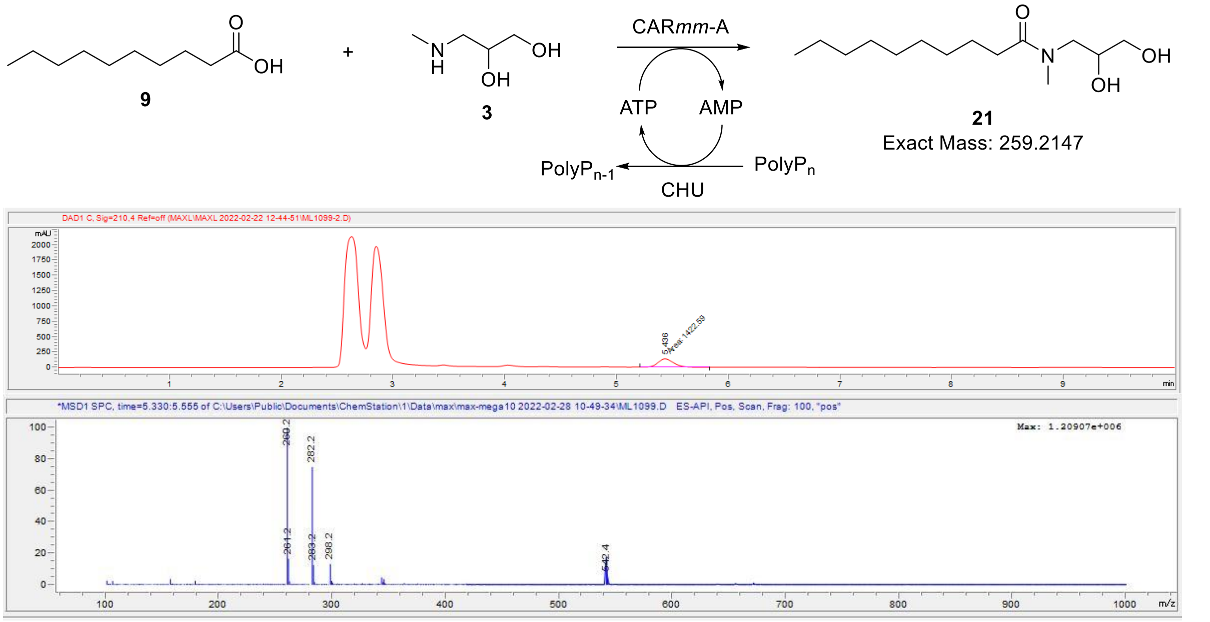

Figure S27: Reaction scheme for the CAR*mm*-A catalysed reaction between **9** and **3** to synthesise **21** (top), LC chromatogram of biotransformation with product peak integrated (middle), and mass spectrum in positive mode of the product peak showing [M+H] = 260, [M+Na] = 282 and [M+K] = 298.

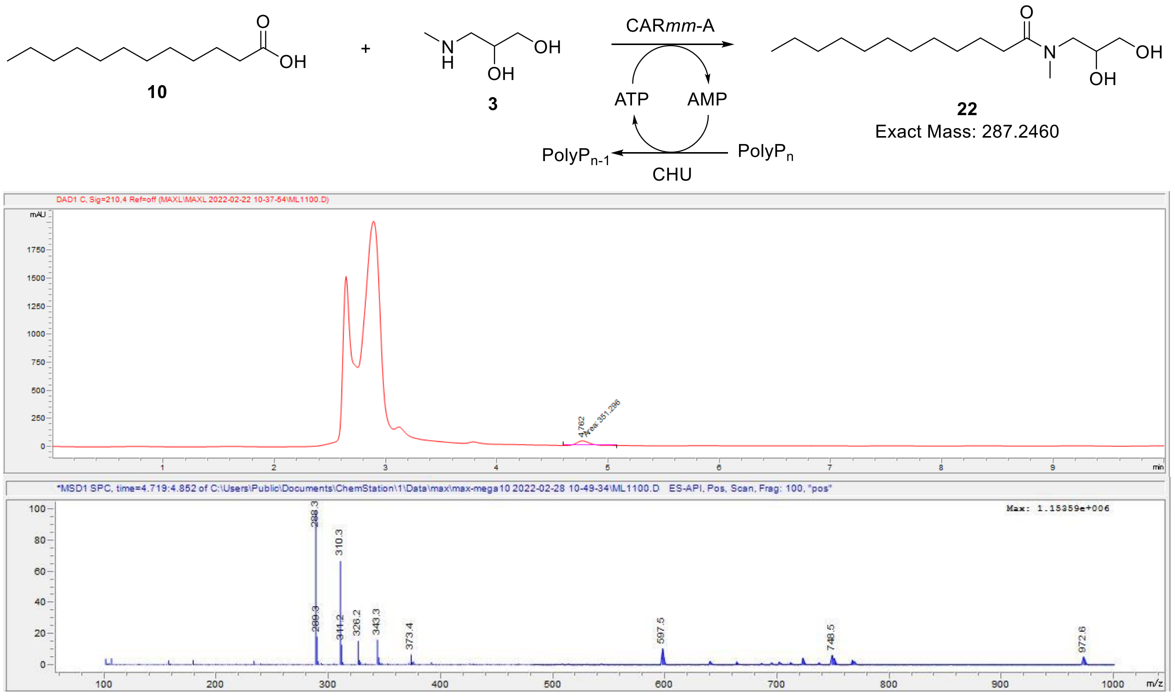

Figure S28: Reaction scheme for the CAR*mm*-A catalysed reaction between **10** and **3** to synthesise **22** (top), LC chromatogram of biotransformation with product peak integrated (middle), and mass spectrum in positive mode of the product peak showing [M+H] = 288, [M+Na] = 310 and [M+K] = 326.

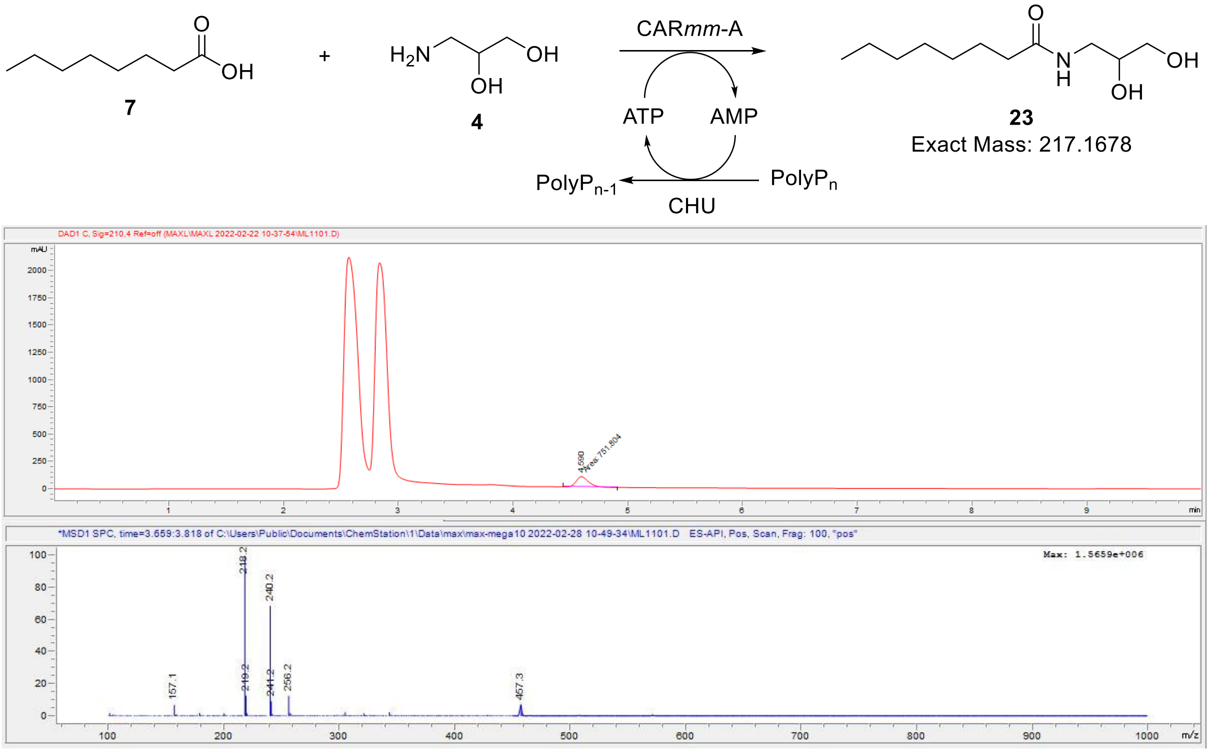

Figure S29: Reaction scheme for the CAR*mm*-A catalysed reaction between **7** and **4** to synthesise **23** (top), LC chromatogram of biotransformation with product peak integrated (middle), and mass spectrum in positive mode of the product peak showing [M+H] = 218, [M+Na] = 240 and [M+K] = 256.

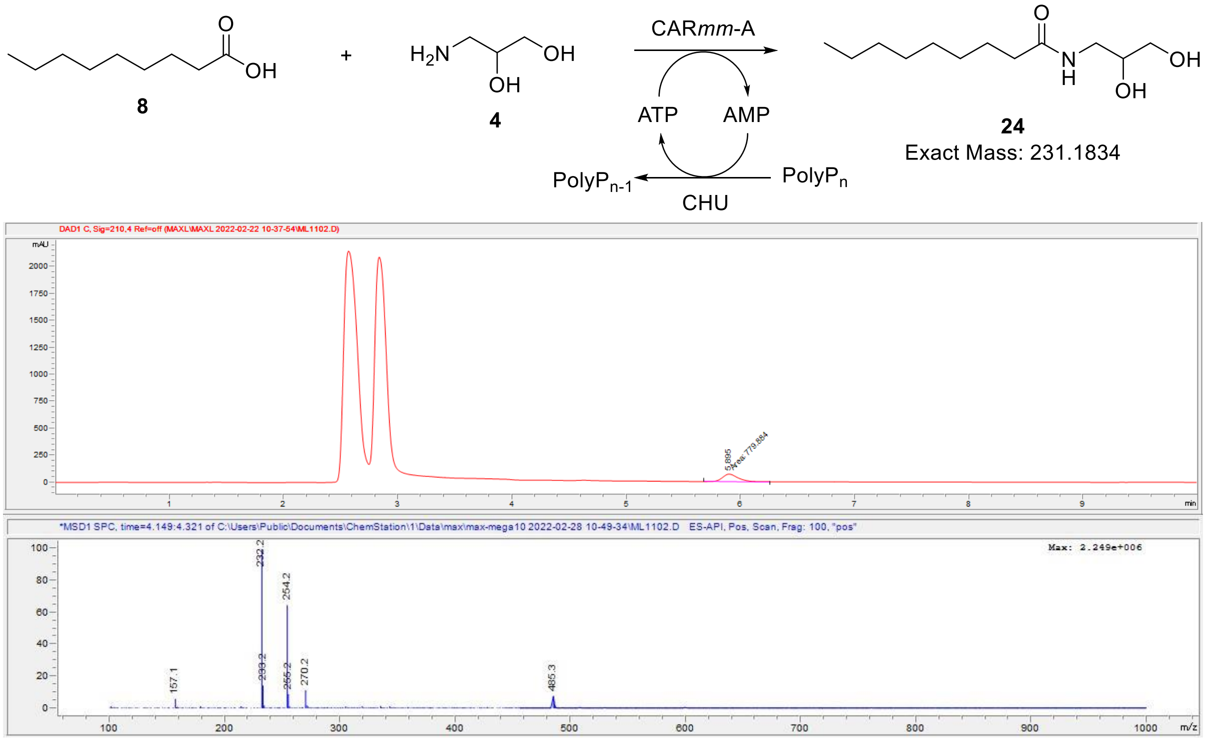

Figure S30: Reaction scheme for the CAR*mm*-A catalysed reaction between **8** and **4** to synthesise **24** (top), LC chromatogram of biotransformation with product peak integrated (middle), and mass spectrum in positive mode of the product peak showing [M+H] = 232, [M+Na] = 254 and [M+K] = 270.

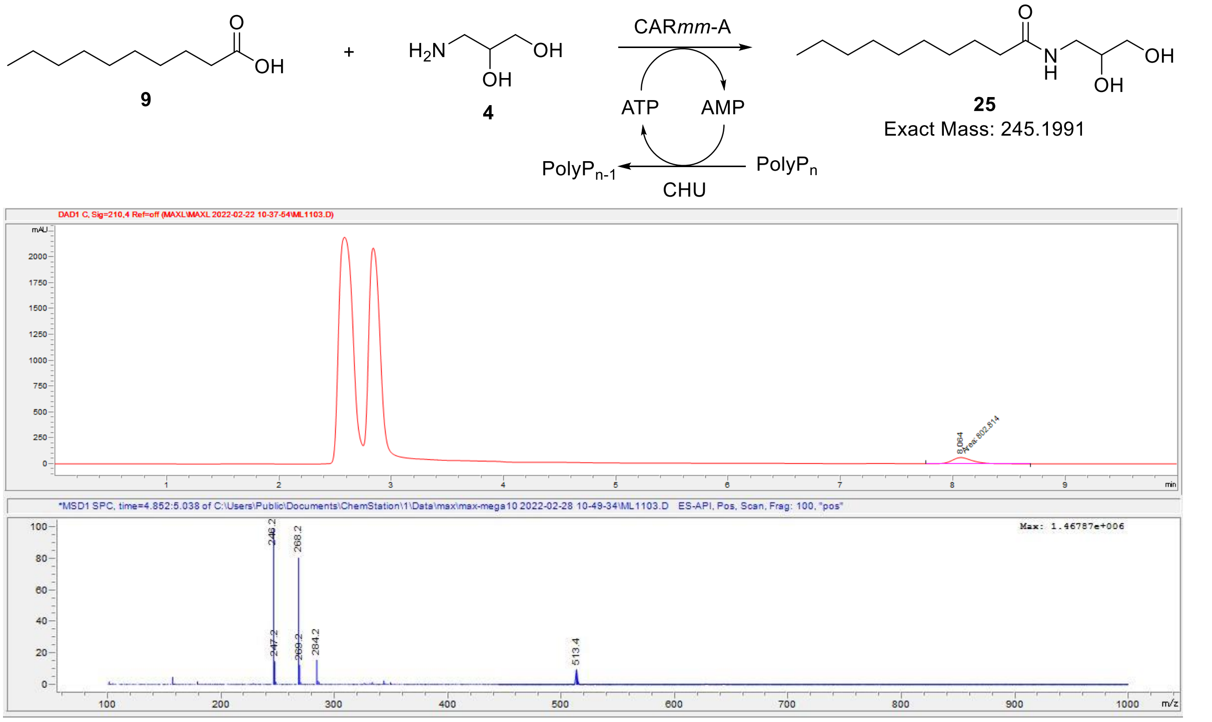

Figure S31: Reaction scheme for the CAR*mm*-A catalysed reaction between **9** and **4** to synthesise **25** (top), LC chromatogram of biotransformation with product peak integrated (middle), and mass spectrum in positive mode of the product peak showing [M+H] = 246, [M+Na] = 268 and [M+K] = 284.

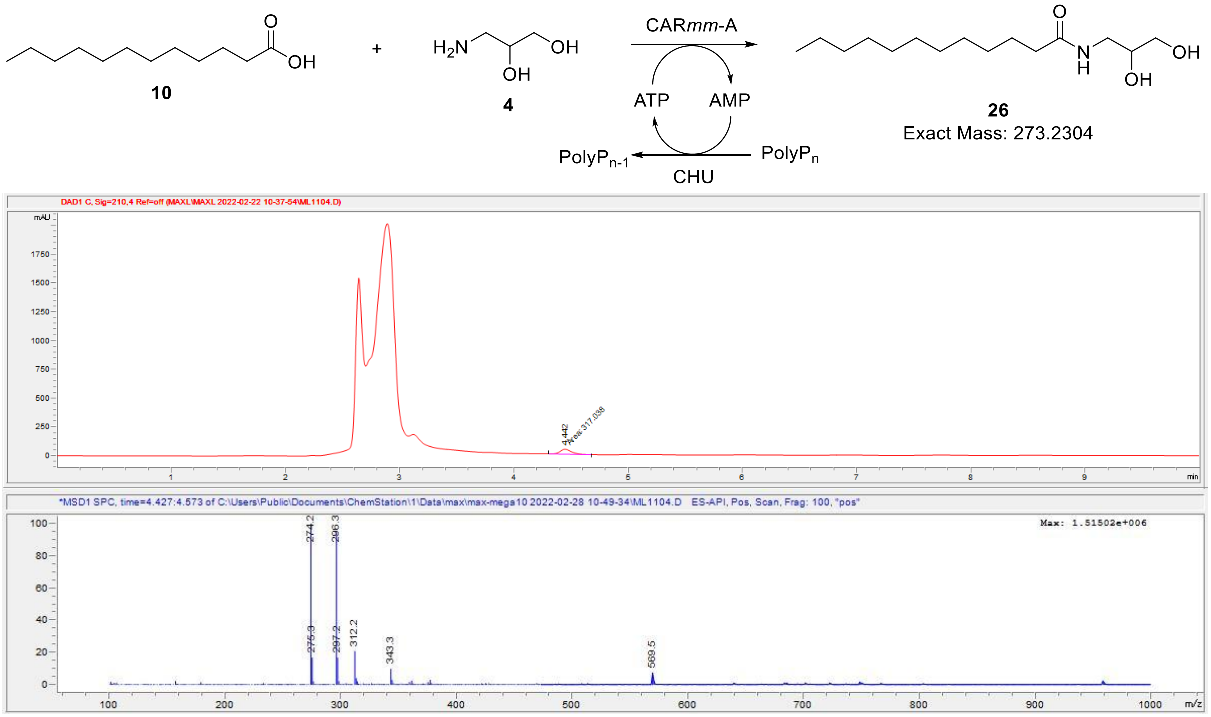

Figure S32: Reaction scheme for the CAR*mm*-A catalysed reaction between **10** and **4** to synthesise **26** (top), LC chromatogram of biotransformation with product peak integrated (middle), and mass spectrum in positive mode of the product peak showing [M+H] = 274, [M+Na] = 296 and [M+K] = 312

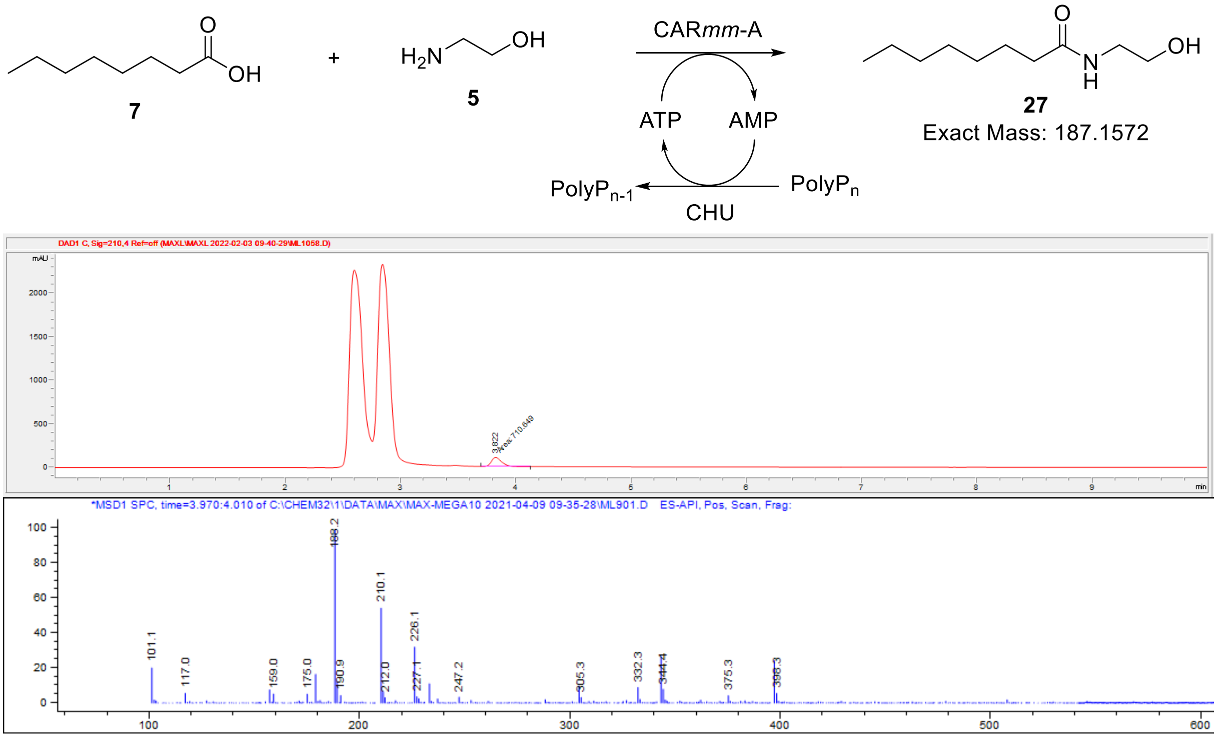

Figure S33: Reaction scheme for the CAR*mm*-A catalysed reaction between **7** and **5** to synthesise **27** (top), LC chromatogram of biotransformation with product peak integrated (middle), and mass spectrum in positive mode of the product peak showing [M+H] = 188, [M+Na] = 210 and [M+K] = 226

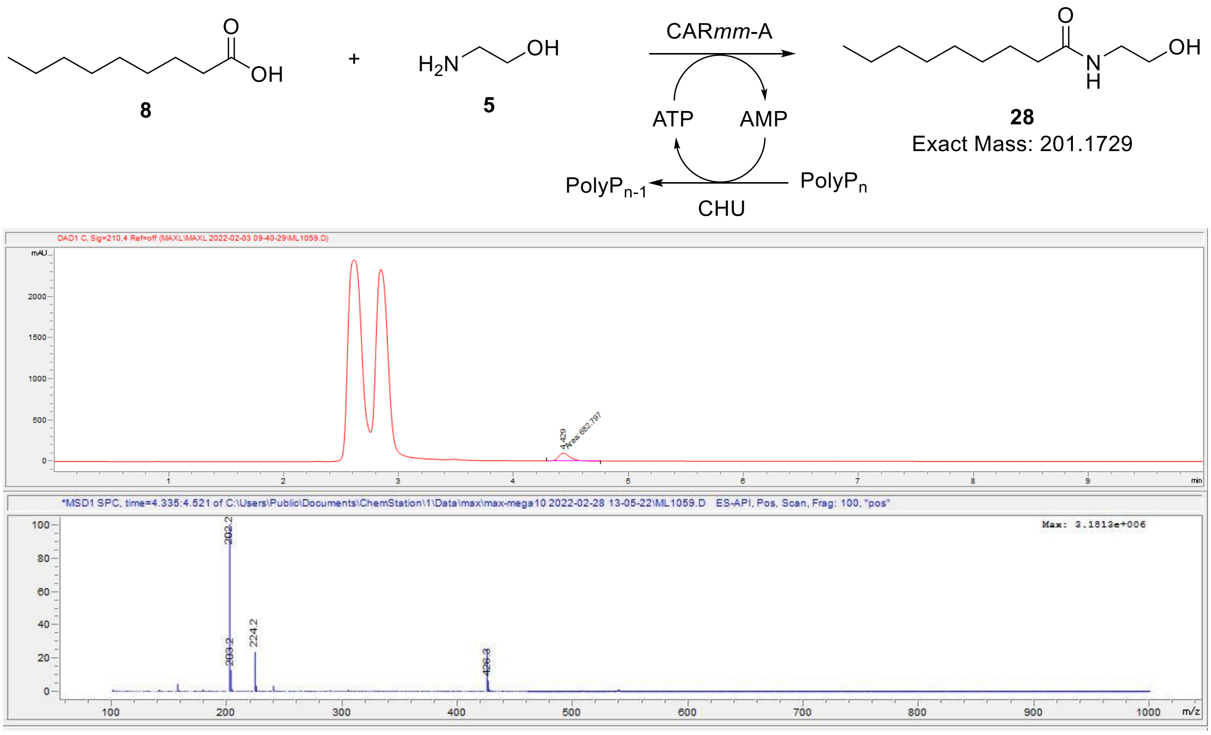

Figure S34: Reaction scheme for the CAR*mm*-A catalysed reaction between **8** and **5** to synthesise **28** (top), LC chromatogram of biotransformation with product peak integrated (middle), and mass spectrum in positive mode of the product peak showing [M+H] = 202 and [M+Na] = 224.

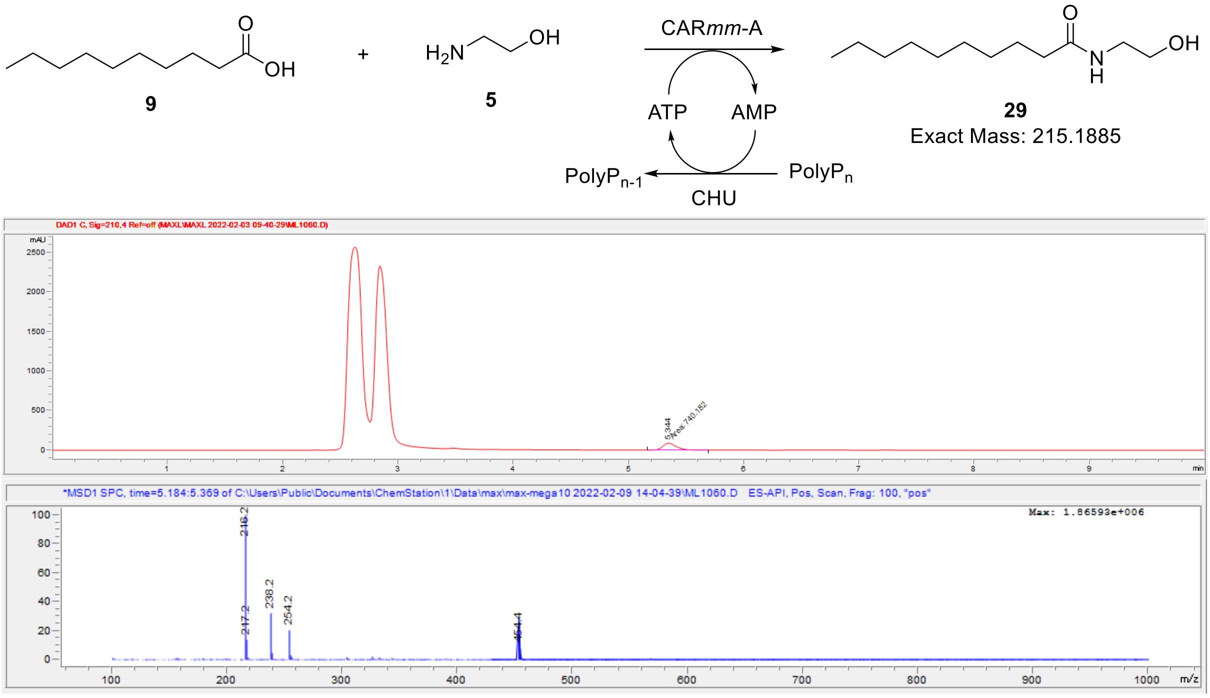

Figure S35: Reaction scheme for the CAR*mm*-A catalysed reaction between **9** and **5** to synthesise **29** (top), LC chromatogram of biotransformation with product peak integrated (middle), and mass spectrum in positive mode of the product peak showing [M+H] = 216, [M+Na] = 238 and [M+K] = 254

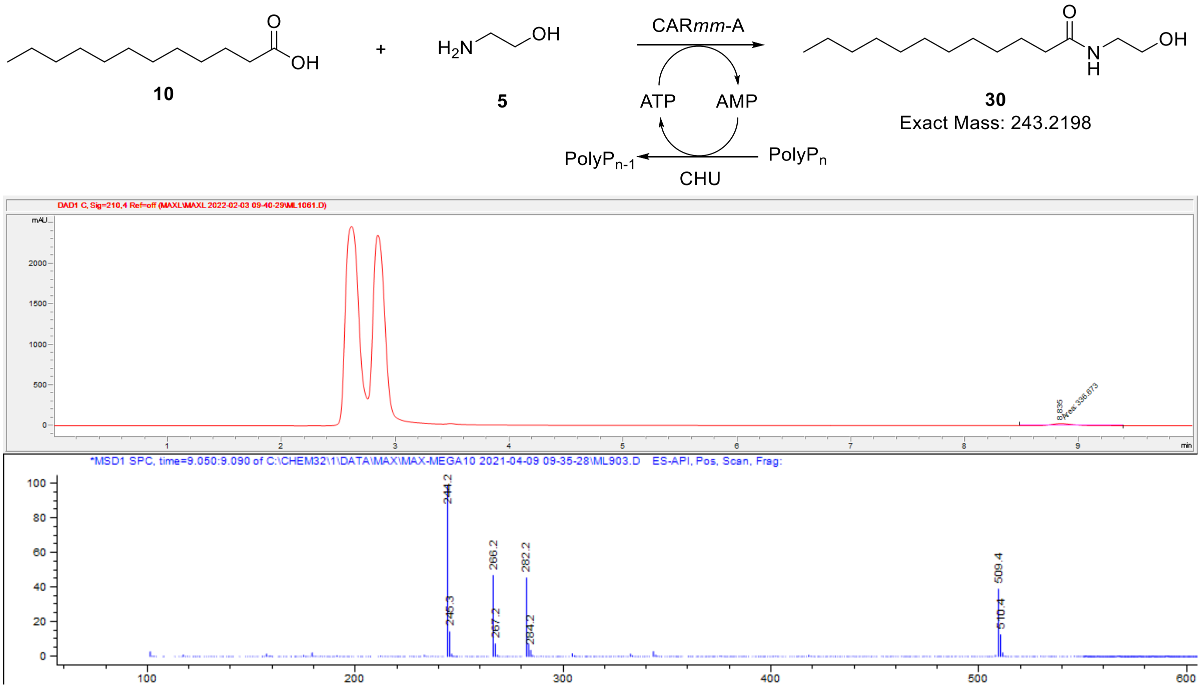

Figure S36: Reaction scheme for the CAR*mm*-A catalysed reaction between **10** and **5** to synthesise **30** (top), LC chromatogram of biotransformation with product peak integrated (middle), and mass spectrum in positive mode of the product peak showing [M+H] = 244, [M+Na] = 266 and [M+K] = 282

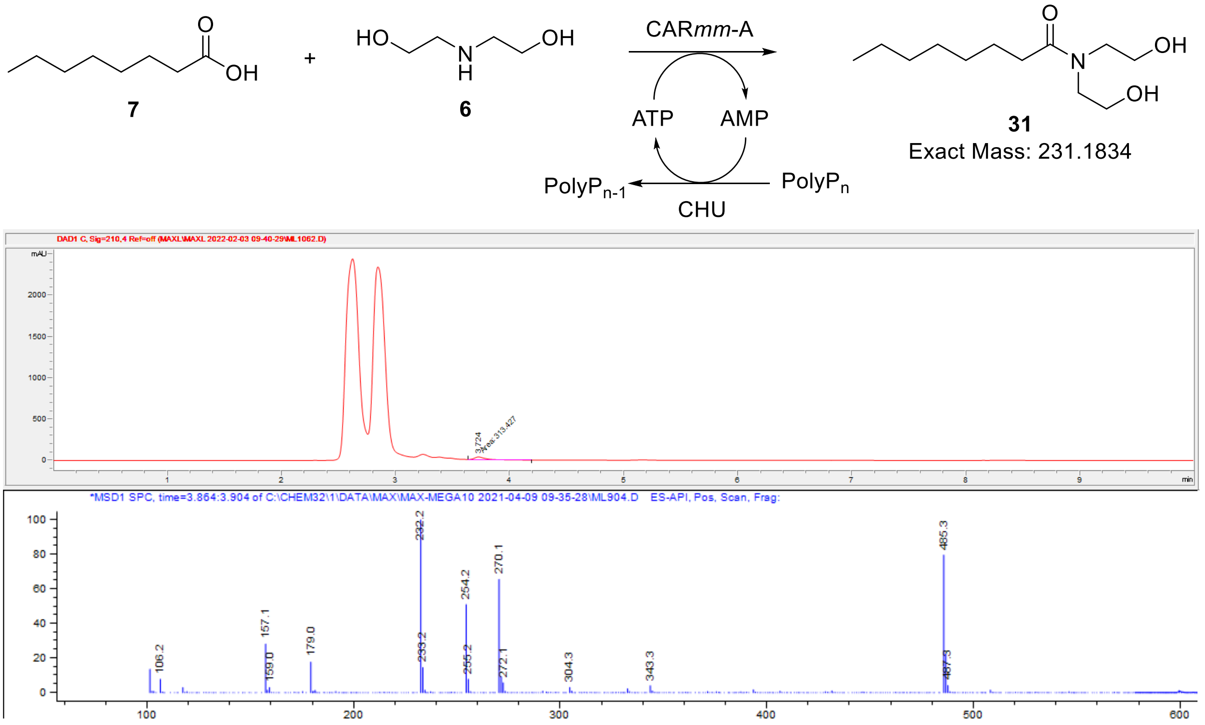

Figure S37: Reaction scheme for the CAR*mm*-A catalysed reaction between **7** and **6** to synthesise **31** (top), LC chromatogram of biotransformation with product peak integrated (middle), and mass spectrum in positive mode of the product peak showing [M+H] = 232, [M+Na] = 254 and [M+K] = 270

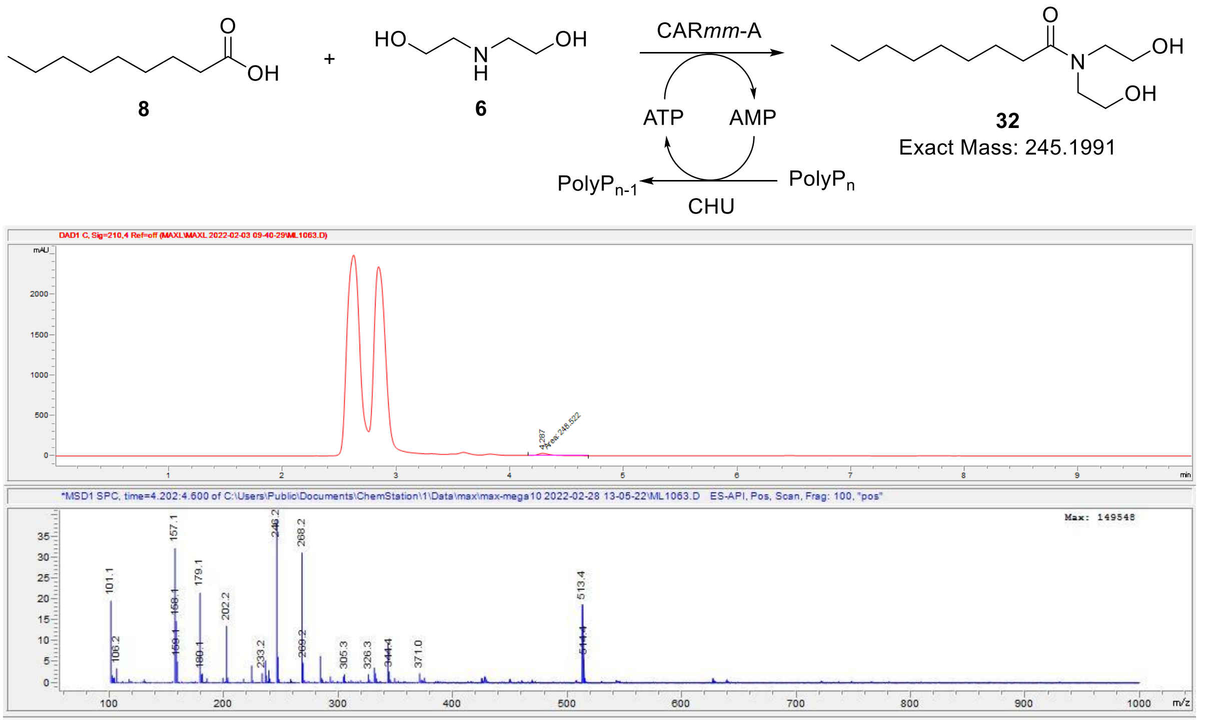

Figure S38: Reaction scheme for the CAR*mm*-A catalysed reaction between **8** and **6** to synthesise **32** (top), LC chromatogram of biotransformation with product peak integrated (middle), and mass spectrum in positive mode of the product peak showing [M+H] = 246 and [M+Na] = 268.

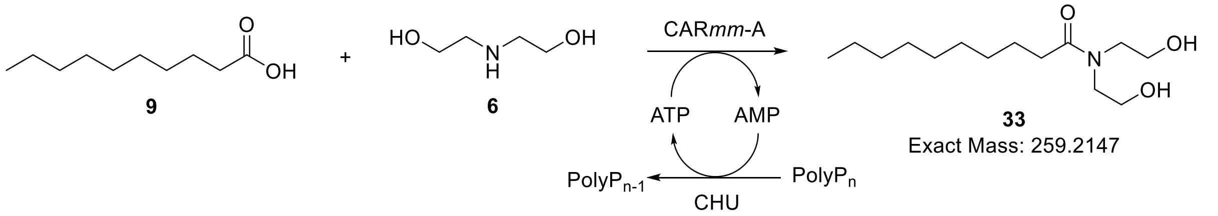

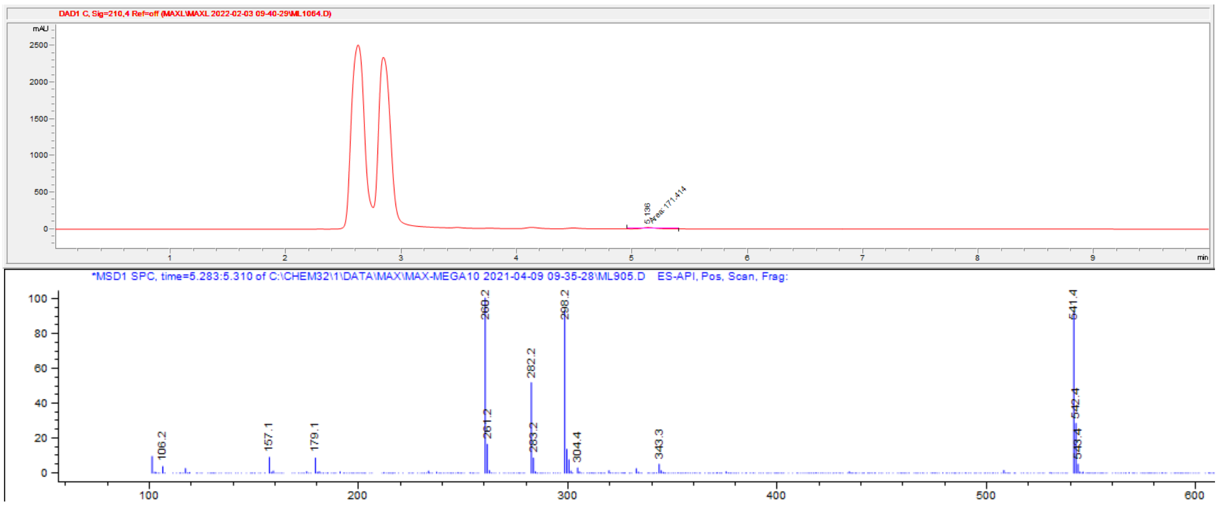

Figure S39: Reaction scheme for the CAR*mm*-A catalysed reaction between **9** and **6** to synthesise **33** (top), LC chromatogram of biotransformation with product peak integrated (middle), and mass spectrum in positive mode of the product peak showing [M+H] = 260, [M+Na] = 282 and [M+K] = 298

Preparative scale reactions

Reaction conditions: Carboxylic acid (5 mM), amine (50 mM), AMP (17.1 mM), MgCl2 (66.5 mM), Polyphosphate (14.9 mg/ml), CHU (13 µM), CAR-A (28 µM) HEPBS buffer (100 mM), 1% DMSO, 30 mL scale, pH 8.5, 37 °C, 250 rpm, 16 h

After 16 hours, the reaction mixture was centrifuged for 5 min at 4000 rpm and the supernatant was freeze-dried. The resulting residue was purified using either normal-phase flash chromatography (DCM/MeOH, Pure C-815 Flash Advanced automated flash chromatography system with UV and ELS detection (Büchi Labortechnik)) or reverse phase flash chromatography (H_2O/MeOH).

MEGA-8 (**11**)

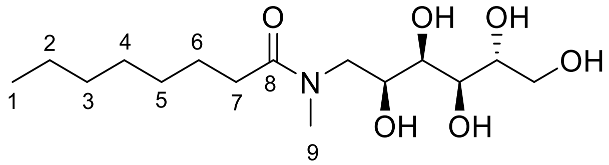

Exact Mass: 321.2151

Isolated yield: 20 mg (42%)

^{1}H NMR (400 MHz, $CDCl_3$) δ 3.95 - 3.31 (m, 8H, C**H**^{sugar}), 3.02 (s, C9**H**), 2.87 (s, C9**H**), 2.35 - 2.18 (m, 2H, C7**H**), 1.51 - 1.46 (m, 2H, C6**H**), 1.26 - 1.19 (m, 8H, C2-5**H**), 0.82 - 0.79 (m, 3H, C1**H**).

Product has a cis- and trans-isomer, as observed by the C10 proton split between two signals (3.02 and 2.87).

^{13}C NMR (101 MHz, $CDCl_3$) δ 175.3 (**C**=O), 73.0 (**C**^{sugar}), 71.8 (**C**^{sugar}), 70.2 (**C**^{sugar}), 63.8 (**C**^{sugar}), 51.7 (**C**^{sugar}), 37.4 (**C**^{sugar}), 33.7 (**C**9H), 31.8 (**C**^{aliph}), 29.7 (**C**^{aliph}), 29.4 (**C**^{aliph}), 29.1 (**C**^{aliph}), 25.0 (**C**^{aliph}), 22.6 (**C**^{aliph}), 14.1 (**C**^{aliph}).

HRMS (ESI+, m/z): calculated for $(C_{15}H_{32}NO_6+)^+$ $[M+H]^+$: 322.2224; found: 322.2217.

N-octanoyl glucamine (**15**)

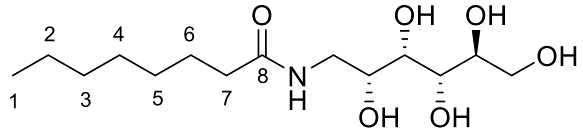

Exact Mass: 307.1995

Isolated yield: 25 mg (54%)

^{1}H NMR (400 MHz, MeOD) δ 8.45 (s, 1H, N**H**^{amide}), 3.73 - 3.33 (m, 7H, C**H**^{sugar}), 3.16 - 3.11 (m, 1H, C**H**^{sugar}), 2.11 (t, J = 7.6 Hz, 2H, C7**H**), 1.57 – 1.43 (m, 2H, C6**H**) 1.25 - 1.19 (m, 8H, C2-5**H**), 0.82 - 0.79 (m, 3H, C1**H**).

^{13}C NMR (101 MHz, MeOD) δ 176.3 (**C**=O), 73.2 (**C**^{sugar}), 72.8 (**C**^{sugar}), 72.4 (**C**^{sugar}), 70.7 (**C**^{sugar}), 64.2 (**C**^{sugar}), 42.8 (**C**^{sugar}), 36.5 (**C**^{aliph}), 32.2 (**C**^{aliph}), 29.7 (**C**^{aliph}), 29.5 (**C**^{aliph}), 26.4 (**C**^{aliph}), 23.0 (**C**^{aliph}), 13.8 (**C**^{aliph}).

HRMS (ESI+, m/z): calculated for $(C_{14}H_{30}NO_6)^+$ [M+H]: 308.2068; found: 308.2061.

N-(2,3-dihydroxypropyl)decanamide (**25**)

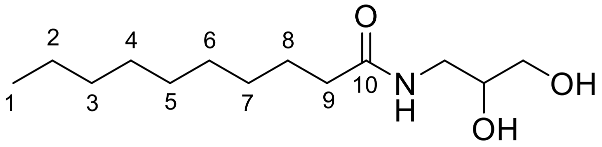

Exact Mass: 245.1991

Isolated yield: 29 mg (78%)

^{1}H NMR (400 MHz, MeOD) δ 8.54 (s, 1H, N**H**^{amide}), 3.72 – 3.60 (m, 1H, C**H**^{glyc}), 3.55 – 3.33 (m, 3H, C**H**^{glyc}), 3.24 – 3.12 (m, 1H, C**H**^{glyc}), 2.20 (t, J = 7.5 Hz, 2H, C9**H**), 1.63 – 1.52 (m, 2H, C8**H**), 1.40 – 1.18 (m, 12H, C2-7**H**), 0.96 – 0.82 (m, 3H, C1**H**).

^{13}C NMR (101 MHz, MeOD) δ 176.3 (**C**=O), 71.5 (**C**^{glyc}), 64.4 (**C**^{glyc}), 42.7 (**C**^{glyc}), 36.4 (**C**^{aliph}), 32.4 (**C**^{aliph}), 30.0 (**C**^{aliph}), 29.8 (**C**^{aliph}), 29.8 (**C**^{aliph}), 29.7 (**C**^{aliph}), 26.4 (**C**^{aliph}), 23.1 (**C**^{aliph}), 13.8 (**C**^{aliph}).

HRMS (ESI+, m/z): calculated for $(C_{13}H_{28}NO_3+)^+$ [M+H]: 246.2064; found: 246.2060.

Gene sequences

All genes contain an N-terminal polyhistidine tag.

CAR*mm*Δ647-1175

ATGAGCCATCATCATCATCATCATGGCACCATGAGCCCGATTACCCGTGAAGAACGTCTGGAAC
GTCGTATTCAGGATCTGTATGCGAACGATCCGCAGTTCGCAGCAGCCAAACCGGCGACCGCGAT
TACCGCGGCGATTGAACGTCCGGGTCTGCCGCTGCCGCAGATCATCGAAACGGTGATGACCGG
CTATGCGGATCGTCCGGCACTGGCACAACGTAGCGTGGAATTTGTGACCGATGCGGGCACCGG
TCATACCACCCTGCGTCTGCTGCCGCATTTTGAAACCATTAGCTATGGCGAACTGTGGGATCGTA
TTAGCGCGCTGGCCGATGTTCTGAGCACCGAACAGACCGTGAAACCGGGCGATCGTGTGTGCC
TGCTGGGCTTTAACAGCGTGGATTATGCGACCATTGATATGACCCTGGCACGTCTGGGTGCTGT
CGCTGTCCCGCTGCAGACCTCTGCTGCGATTACCCAGCTGCAGCCGATTGTGGCGGAAACCCA
GCCGACCATGATTGCGGCGAGCGTGGATGCCCTGGCCGATGCGACCGAACTGGCACTGAGTGG
TCAAACGGCTACGCGTGTGCTGGTGTTTGATCATCATCGTCAGGTGGATGCGCATCGTGCGGCG
GTTGAAAGCGCGCGTGAACGTCTGGCCGGTAGCGCGGTGGTTGAAACCCTGGCCGAAGCGATT
GCGCGTGGTGATGTGCCGCGTGGTGCGAGCGCGGGTAGCGCACCGGGCACCGATGTGAGCGA
TGATAGCCTGGCCCTGCTGATTTATACCTCTGGTAGTACGGGTGCGCCGAAAGGCGCCATGTAT
CCGCGTCGTAACGTGGCGACCTTTTGGCGTAAACGTACCTGGTTTGAAGGCGGCTATGAACCGA
GCATTACCCTGAACTTTATGCCGATGAGCCATGTGATGGGCCGTCAGATTCTGTATGGCACCCT
GTGCAACGGCGGCACCGCGTATTTTGTGGCGAAAAGCGATCTGAGCACCCTGTTTGAAGATCTG
GCCCTGGTGCGTCCGACCGAACTGACCTTCGTCCCGCGTGTTTGGGATATGGTGTTCGATGAAT
TTCAGAGCGAAGTGGATCGTCGTCTGGTGGATGGCGCGGATCGTGTTGCGCTGGAAGCGCAGG
TGAAAGCGGAAATTCGTAACGATGTGCTGGGCGGTCGTTATACCTCTGCTCTGACGGGTTCTGC
TCCGATTAGCGATGAAATGAAAGCGTGGGTGGAAGAACTGCTGGATATGCATCTGGTGGAAGGC
TATGGCAGCACCGAAGCGGGCATGATTCTGATTGATGGCGCGATTCGTCGTCCGGCGGTGCTG
GATTATAAACTGGTGGATGTTCCGGATCTGGGCTATTTTCTGACCGATCGTCCGCATCCGCGTG
GCGAACTGCTGGTGAAAACCGATAGCCTGTTTCCGGGCTATTATCAGCGTGCGGAAGTGACCGC
GGATGTGTTTGATGCGGATGGCTTTTATCGCACCGGCGATATTATGGCGGAAGTGGGCCCGGAA
CAGTTTGTGTATCTGGATCGTCGTAACAACGTGCTGAAACTGAGCCAGGGCGAATTTGTTACCGT
GAGCAAACTGGAAGCGGTGTTTGGCGATAGCCCGCTGGTGCGTCAGATTTATATTTATGGCAAC
AGCGCGCGTGCGTATCTGCTGGCCGTGATTGTGCCGACCCAGGAAGCGCTGGACGCGGTCCC
GGTTGAAGAACTGAAAGCGCGTCTGGGTGACTCTCTGCAGGAAGTGGCGAAAGCGGCGGGTCT
GCAGAGCTATGAAATTCCGCGCGATTTTATTATCGAAACCACCCCGTGGACCCTGGAAAACGGC
CTGCTGACGGGTATTCGTAAACTGGCCCGTCCGCAGCTGAAAAAACATTATGGTGAACTGCTGG
AACAAATTTATACCGATCTGGCCCACGGCCAGGCGGATGAACTGCGTAGCCTGCGTCAGAGCTA
A

CHU

ATGGGCAGCAGCCATCATCATCATCATCACAGCAGCGGCCTGGTGCCGCGCGGCAGCCATATG
GCAACCGATTTTAGCAAACTGAGCAAATATGTTGAAACCCTGCGTGTTAAACCGAAACAGAGCAT
TGATCTGAAAAAAGATTTCGACACCGACTACGATCATAAAATGCTGACCAAAGAAGAAGGCGAAG
AACTGCTGAATCTGGGTATTTCAAAACTGAGCGAGATCCAAGAAAAACTGTATGCAAGCGGCACC
AAAAGCGTTCTGATTGTTTTTCAGGCAATGGATGCAGCAGGTAAAGATGGCACCGTTAAACATAT
TATGACCGGTCTGAATCCGCAGGGTGTTAAAGTTACCAGCTTTAAAGTTCCGAGCAAAATCGAAC
TGAGCCATGATTATCTGTGGCGTCATTATGTTGCACTGCCTGCAACCGGTGAAATTGGTATCTTT
AATCGTAGCCACTATGAAAATGTTCTGGTTACCCGTGTTCATCCGGAATATCTGCTGAGCGAACA
GACCAGCGGTGTTACCGCAATTGAACAGGTTAATCAGAAATTCTGGGATAAACGCTTTCAGCAGA
TCAACAACTTTGAACAGCATATTAGCGAAAACGGCACCATTGTGCTGAAATTCTTTCTGCATGTGA
GCAAGAAAGAGCAGAAAAAACGCTTTATTGAACGCATCGAACTGGACACCAAAAACTGGAAATTT
AGCACAGGCGATCTGAAAGAACGTGCACATTGGAAAGATTATCGCAACGCCTATGAAGATATGCT
GGCAAATACCAGCACCAAACAGGCACCGTGGTTTGTTATTCCGGCAGATGATAAATGGTTTACCC
GTCTGCTGATTGCCGAAATTATCTGTACCGAACTGGAAAAACTGAATCTGACCTTTCCGACCGTT
AGCCTGGAACAGAAAGCAGAGCTGGAAAAAGCAAAAGCAGAACTGGTTGCCGAAAAAAGCAGC
GATTAA

References

[1] M. Lubberink, C. Schnepel, J. Citoler, S. R. Derrington, W. Finnigan, M. A. Hayes, N. J. Turner, S. L. Flitsch, *ACS Catal.* **2020**, *10*, 10005–10009.

Compound spectra

20220301-1119-B400_MIB-16.10.fid
Ref ML 1107
Group Turner_N
H1_Day CDCl3 /mnt/nmrdata/Turner_N mbdxjlp2 16

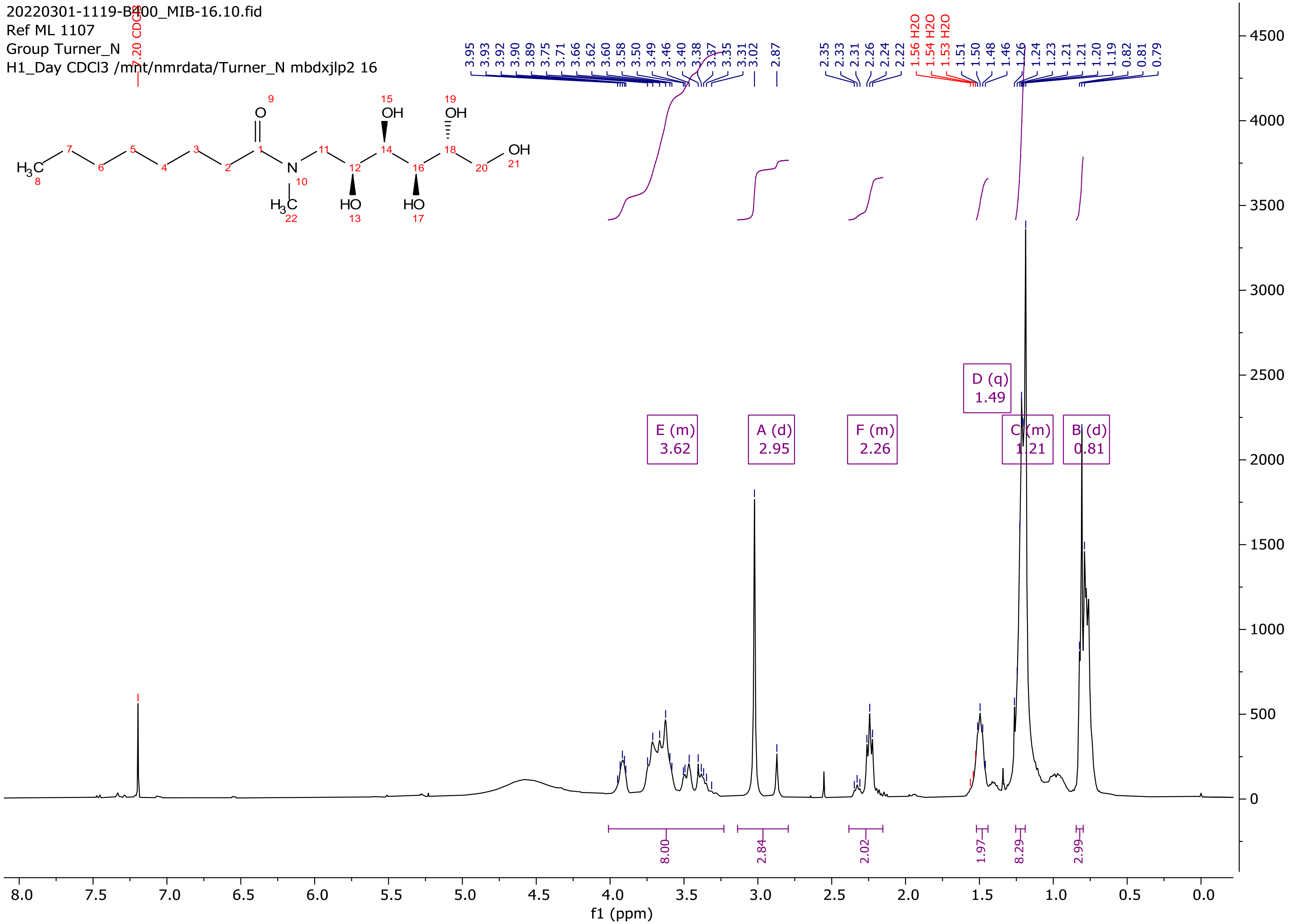

20220301-1204-B400_MIB-17.10.fid
Ref ML1107
Group Flitsch_S
C13_CPD_Night256 CDCl3 /mnt/nmrdata/Flitsch_S u67644ml 17

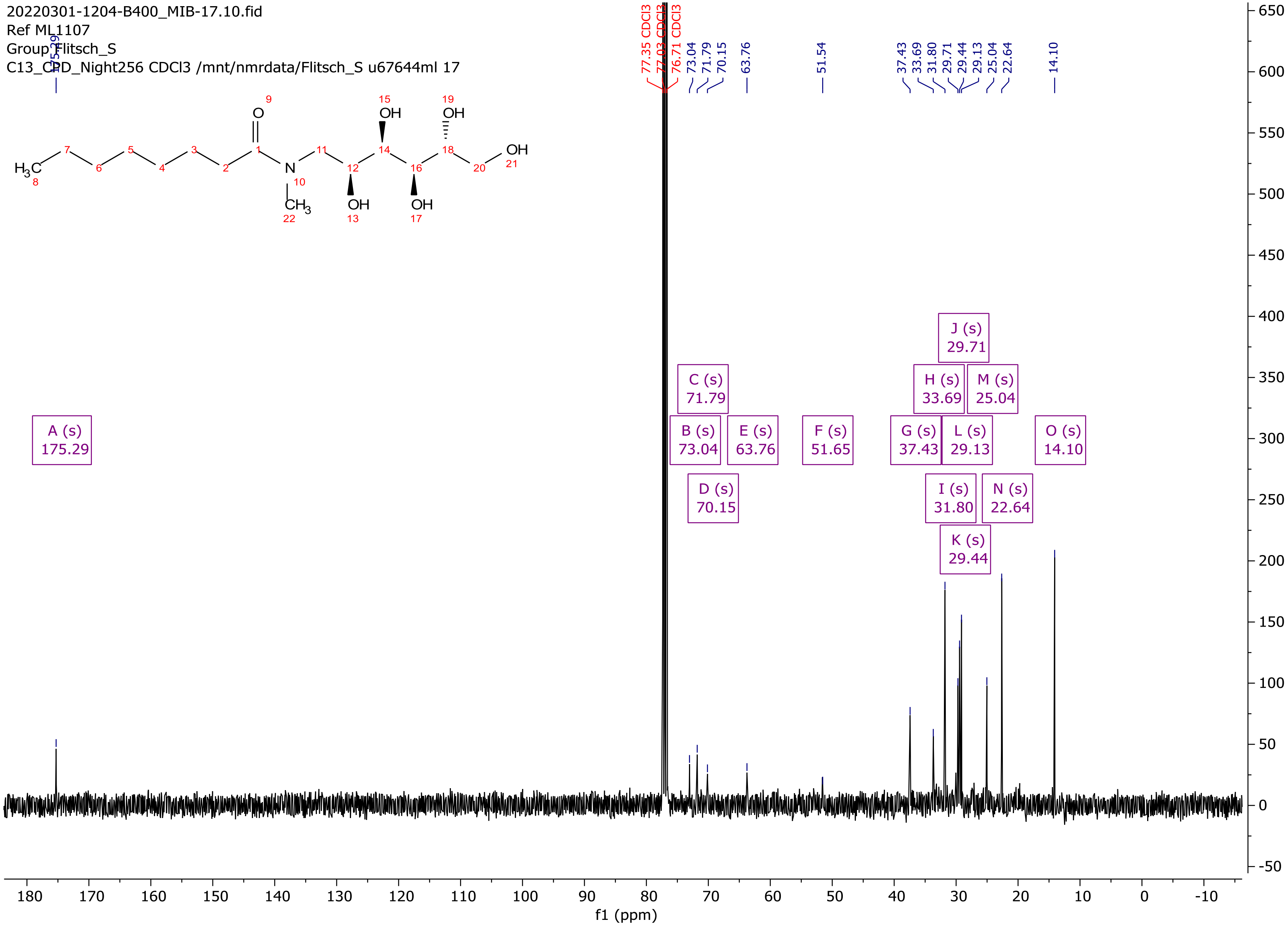

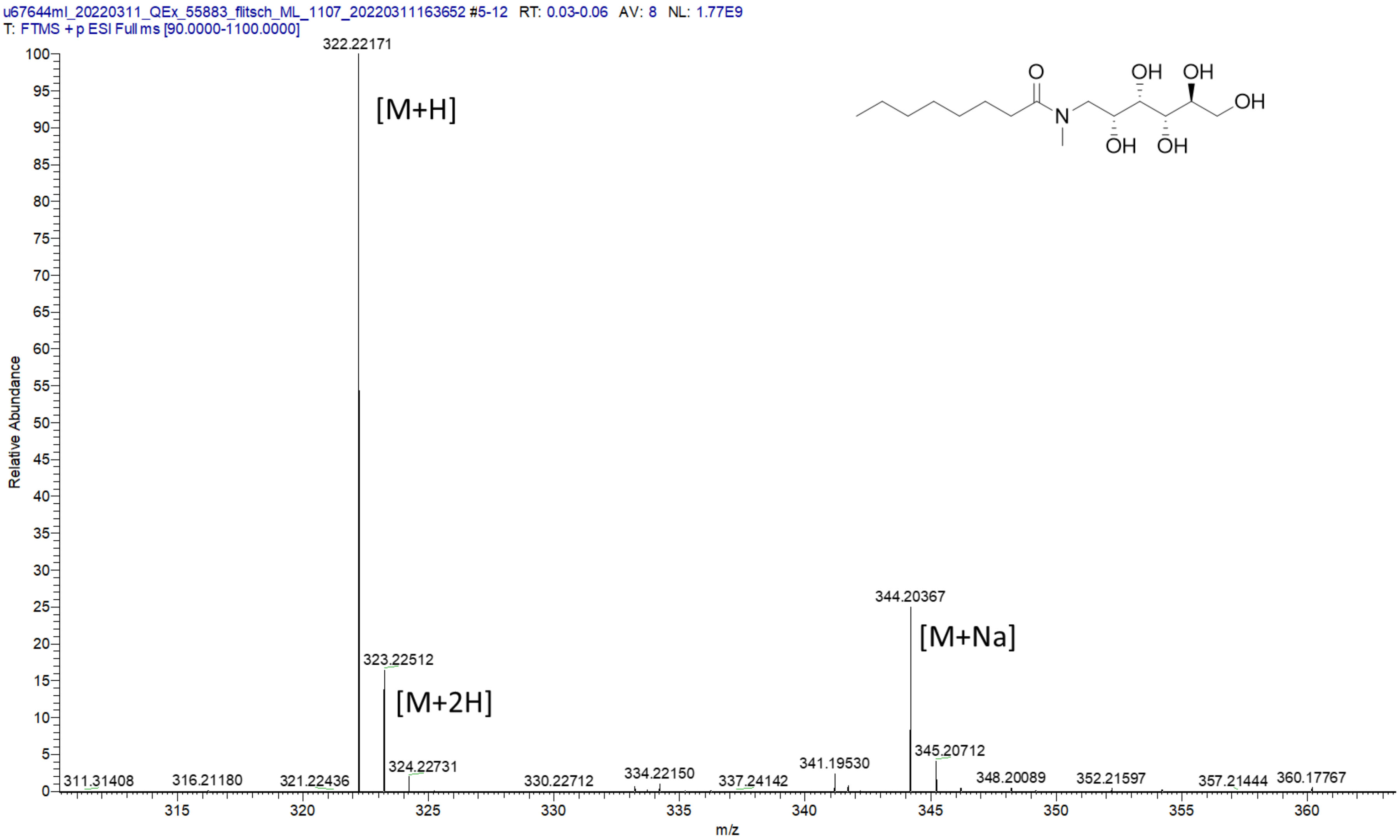
u67644ml_20220311_QEx_55883_flitsch_ML_1107_20220311163652 #5-12 RT: 0.03-0.06 AV: 8 NL: 1.77E9
T: FTMS + p ESI Full ms [90.0000-1100.0000]
322.22171
[M+H]
323.22512
[M+2H]
324.22731
344.20367
[M+Na]
345.20712
311.31408
316.21180
321.22436
330.22712
334.22150
337.24142
341.19530
348.20089
352.21597
357.21444
360.17767
Relative Abundance
m/z
O
OH
OH
OH
N
OH
OH

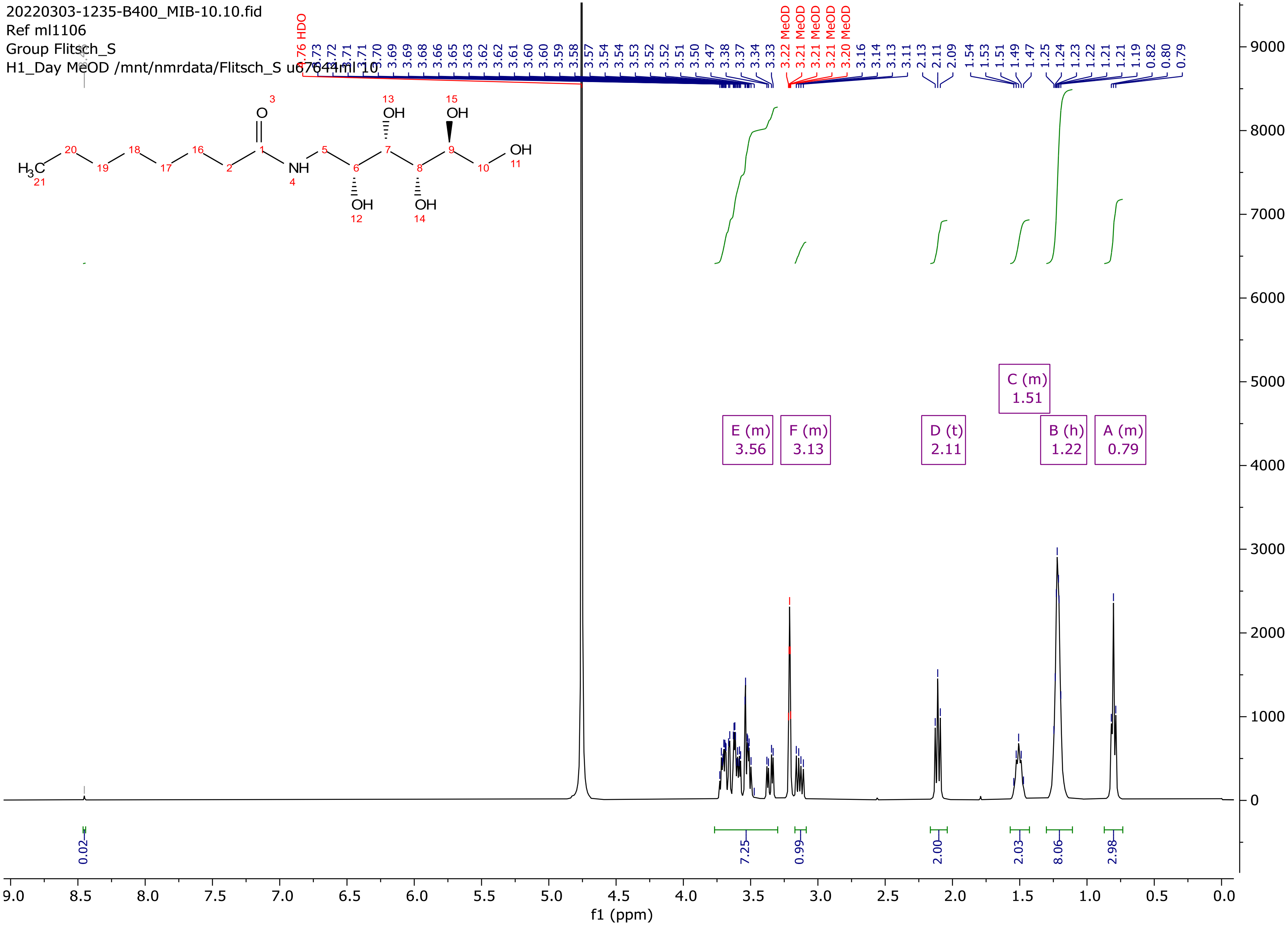

20220303-1235-B400_MIB-10.10.fid
Ref ml1106
Group Flitsch_S
H1_Day MeOD /mnt/nmrdata/Flitsch_S u67644ml 10
E (m) 3.56
F (m) 3.13
D (t) 2.11
C (m) 1.51
B (h) 1.22
A (m) 0.79
f1 (ppm)

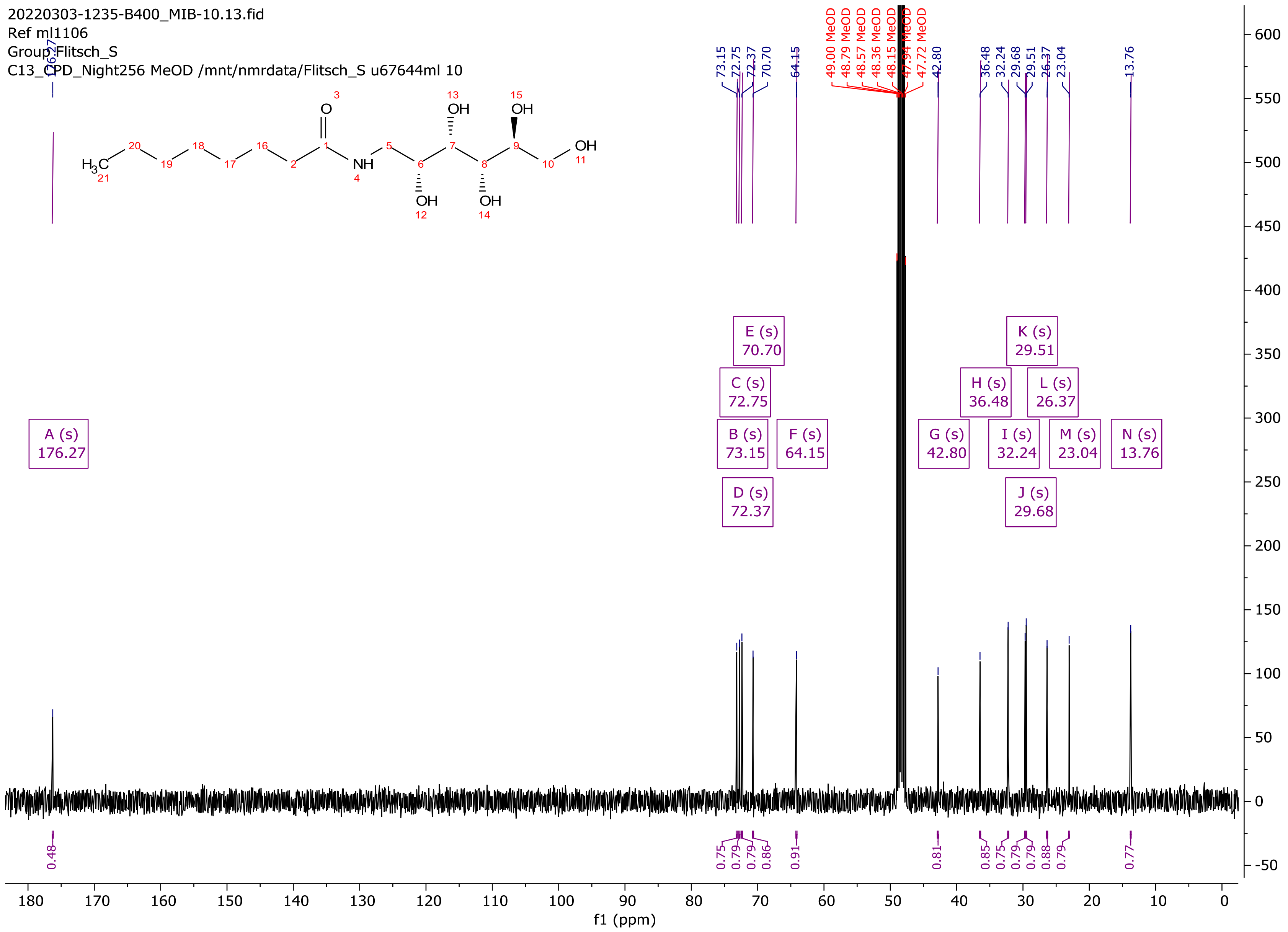

20220303-1235-B400_MIB-10.13.fid
Ref ml1106
Group Flitsch_S
C13_CPD_Night256 MeOD /mnt/nmrdata/Flitsch_S u67644ml 10
176.27
73.15
72.75
72.37
70.70
64.15
49.00 MeOD
48.79 MeOD
48.57 MeOD
48.36 MeOD
48.15 MeOD
47.94 MeOD
47.72 MeOD
42.80
36.48
32.24
29.68
29.51
26.37
23.04
13.76
A (s) 176.27
B (s) 73.15
C (s) 72.75
D (s) 72.37
E (s) 70.70
F (s) 64.15
G (s) 42.80
H (s) 36.48
I (s) 32.24
J (s) 29.68
K (s) 29.51
L (s) 26.37
M (s) 23.04
N (s) 13.76
f1 (ppm)

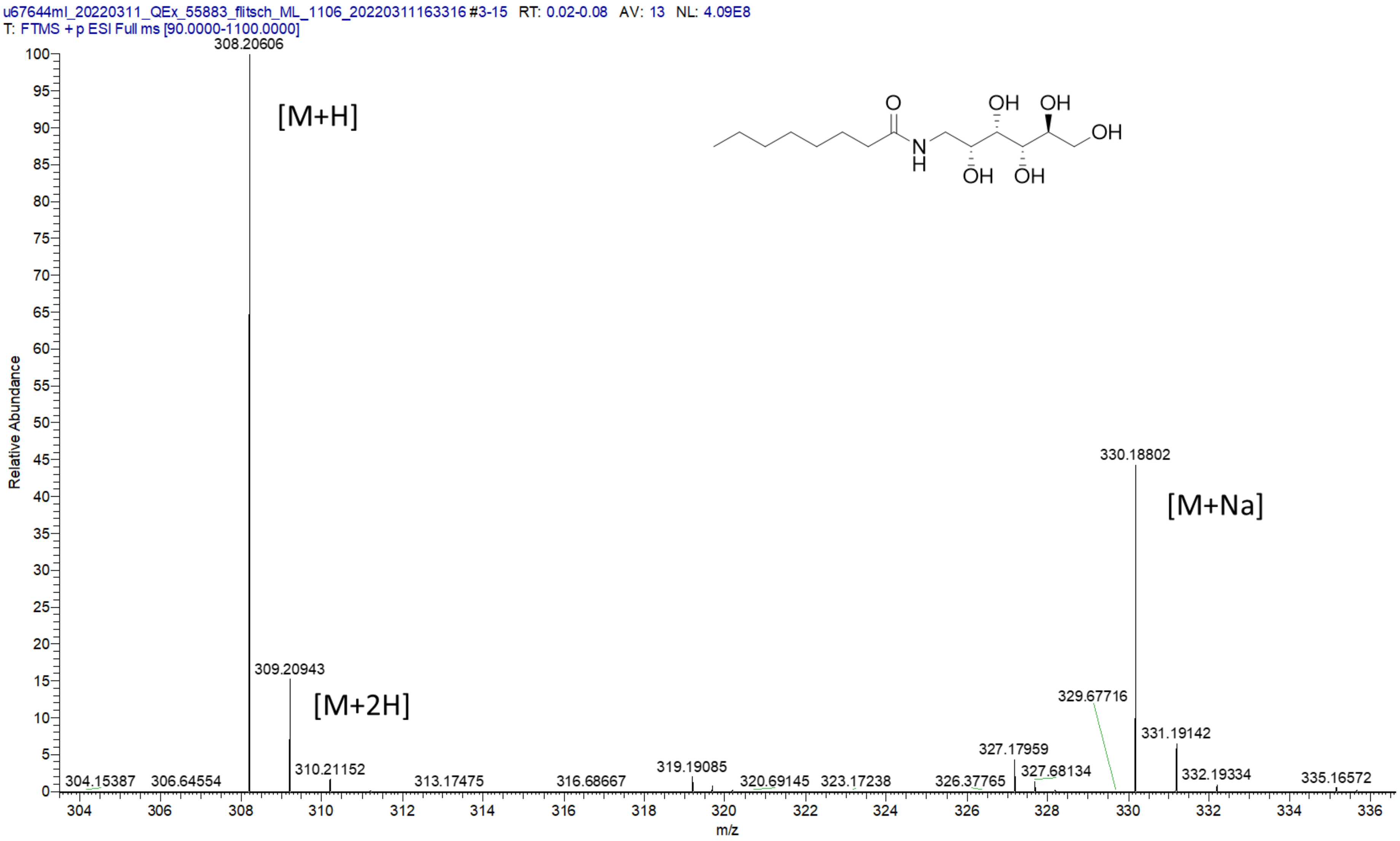
u67644ml_20220311_QEx_55883_flitsch_ML_1106_20220311163316 #3-15 RT: 0.02-0.08 AV: 13 NL: 4.09E8
T: FTMS + p ESI Full ms [90.0000-1100.0000]
308.20606
[M+H]
309.20943
[M+2H]
330.18802
[M+Na]
329.67716
331.19142
327.17959
327.68134
304.15387
306.64554
310.21152
313.17475
316.68667
319.19085
320.69145
323.17238
326.37765
332.19334
335.16572
Relative Abundance
m/z
O
OH
OH
OH
N
H
OH
OH

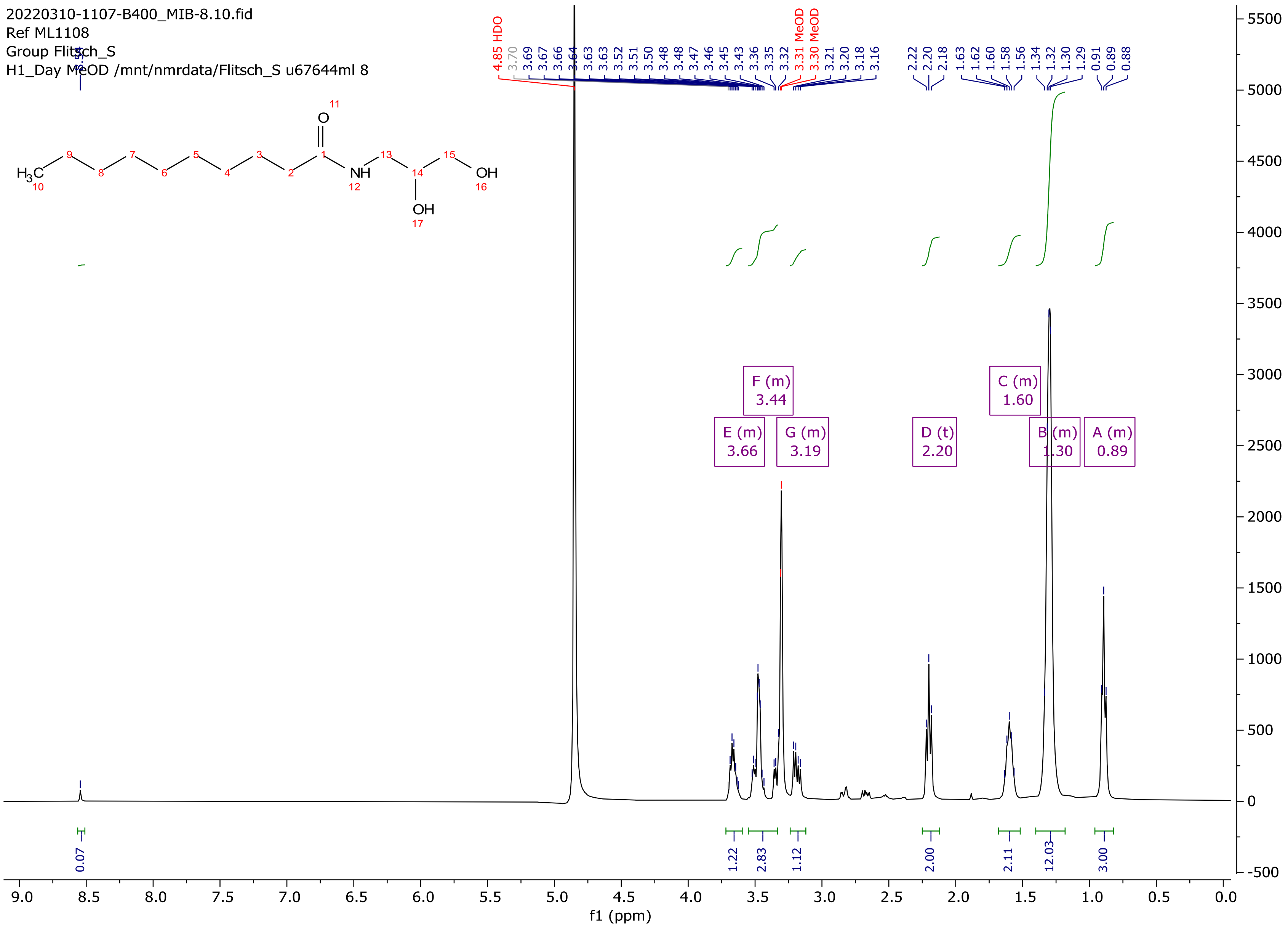
20220310-1107-B400_MIB-8.10.fid
Ref ML1108
Group Flitsch_S
H1_Day MeOD /mnt/nmrdata/Flitsch_S u67644ml 8
4.85 HDO
3.31 MeOD
3.30 MeOD
E (m) 3.66
F (m) 3.44
G (m) 3.19
D (t) 2.20
C (m) 1.60
B (m) 1.30
A (m) 0.89
0.07
1.22
2.83
1.12
2.00
2.11
12.03
3.00
f1 (ppm)

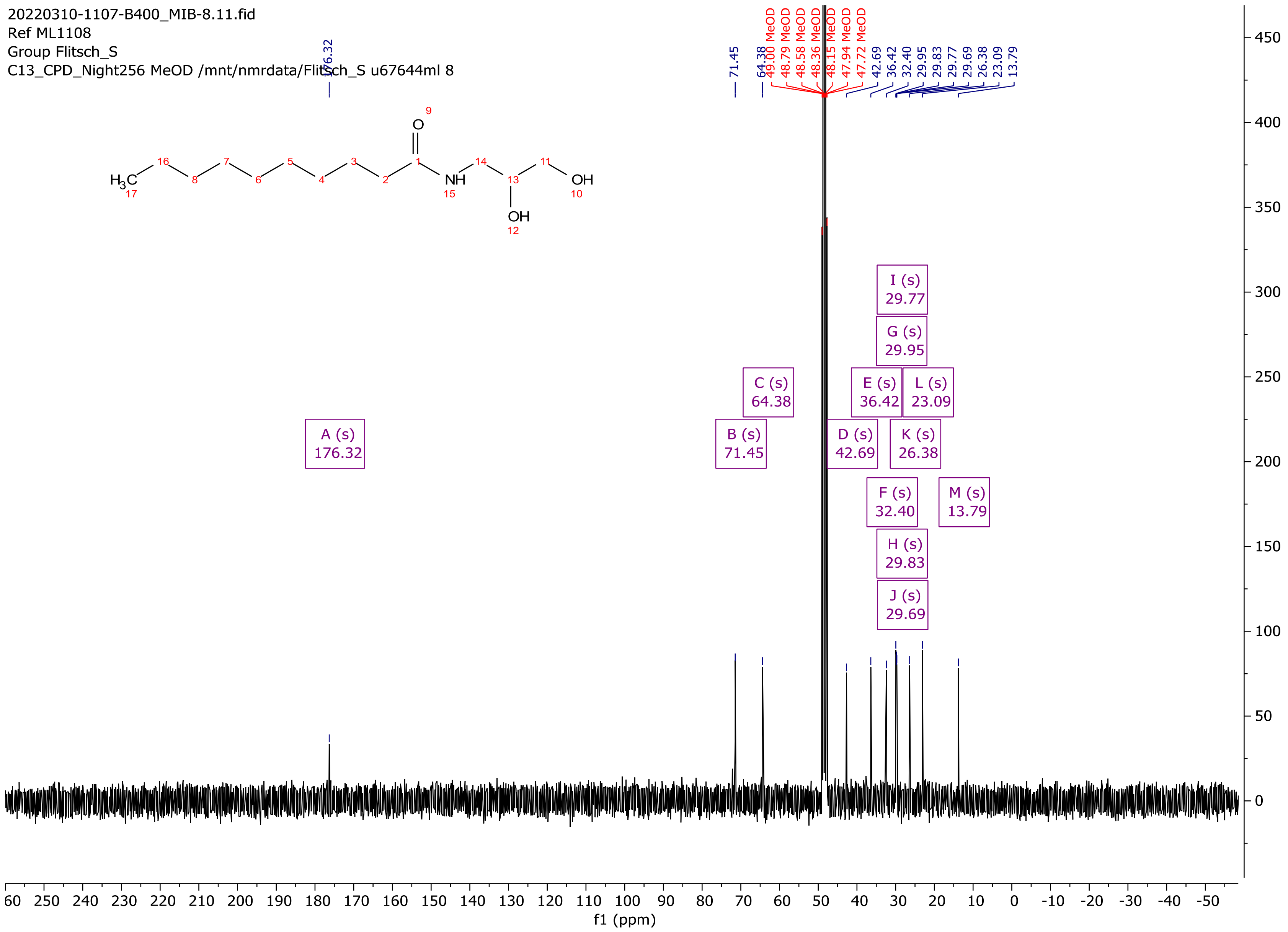

20220310-1107-B400_MIB-8.11.fid
Ref ML1108
Group Flitsch_S
C13_CPD_Night256 MeOD /mnt/nmrdata/Flitsch_S u67644ml 8
176.32
71.45
64.38
49.00 MeOD
48.79 MeOD
48.58 MeOD
48.36 MeOD
48.15 MeOD
47.94 MeOD
47.72 MeOD
42.69
36.42
32.40
29.95
29.83
29.77
29.69
26.38
23.09
13.79
A (s) 176.32
B (s) 71.45
C (s) 64.38
D (s) 42.69
E (s) 36.42
F (s) 32.40
G (s) 29.95
H (s) 29.83
I (s) 29.77
J (s) 29.69
K (s) 26.38
L (s) 23.09
M (s) 13.79
f1 (ppm)

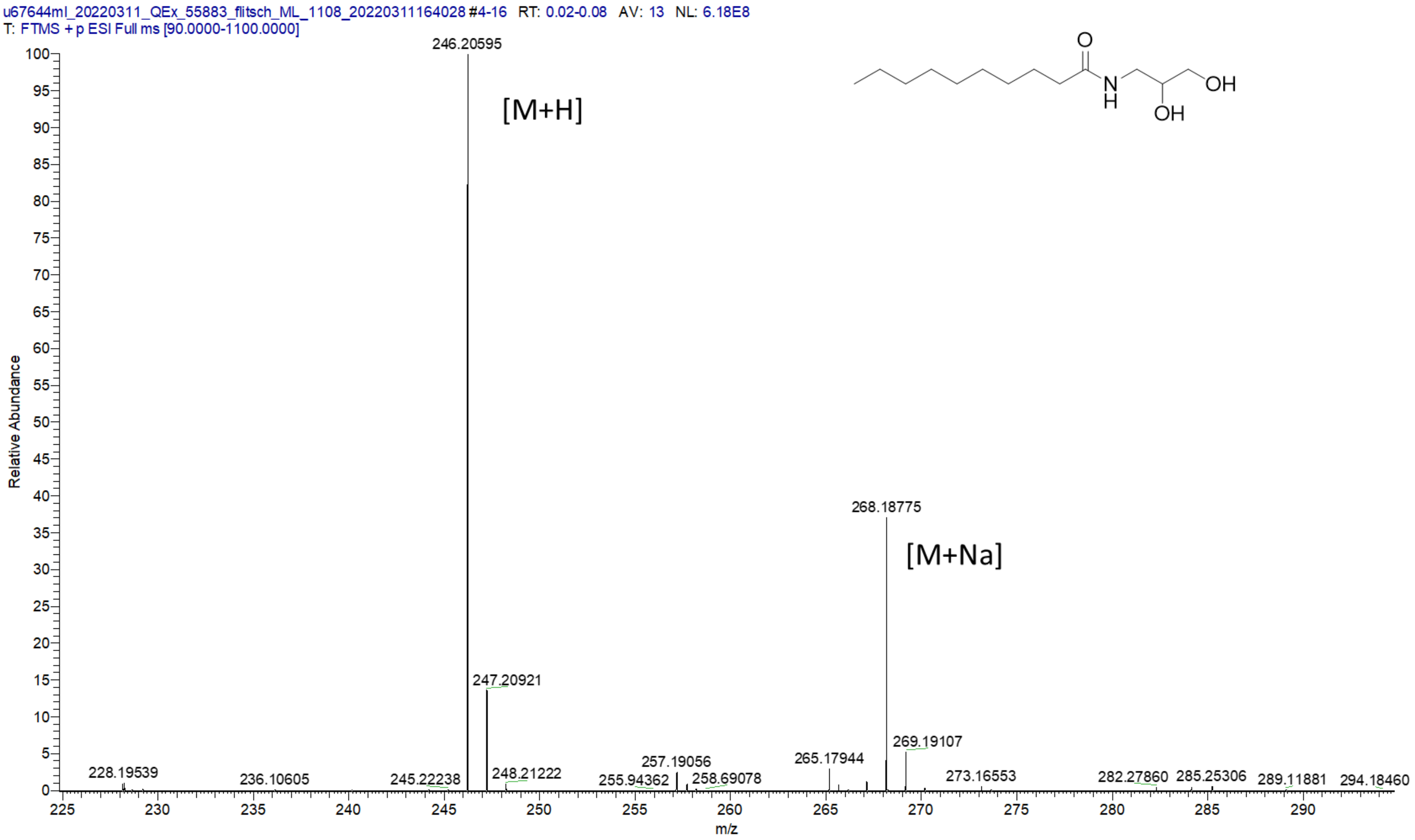
u67644ml_20220311_QEx_55883_flitsch_ML_1108_20220311164028 #4-16 RT: 0.02-0.08 AV: 13 NL: 6.18E8
T: FTMS + p ESI Full ms [90.0000-1100.0000]
246.20595
[M+H]
268.18775
[M+Na]
247.20921
269.19107
228.19539
236.10605
245.22238
248.21222
255.94362
257.19056
258.69078
265.17944
273.16553
282.27860
285.25306
289.11881
294.18460
Relative Abundance
m/z
O
N
H
OH
OH
